# Identification of simple sequence repeat markers linked to heat tolerance in rice using bulked segregant analysis in F_2_ population of NERICA-L 44 × Uma

**DOI:** 10.3389/fpls.2023.1113838

**Published:** 2023-03-27

**Authors:** K. Stephen, K. Aparna, R. Beena, R. P. Sah, Uday Chand Jha, Sasmita Behera

**Affiliations:** ^1^ Department of Plant Physiology, College of Agriculture, Vellayani, Kerala Agricultural University, Thiruvananthapuram, India; ^2^ Crop Improvement Division, Indian Council of Agricultural Research (ICAR)-National Rice Research Institute, Cuttack, India; ^3^ Crop Improvement Division, Indian Institute of Pulses Research, Kanpur, India

**Keywords:** Bulked Segregant Analysis (BSA), heat tolerance, SSR markers, NERICA-L 44, rice physiology, gene annotation

## Abstract

The damage caused by high temperature is one of the most important abiotic stress affecting rice production. Reproductive stage of rice is highly susceptible to high temperature. The present investigation was undertaken to identify polymorphic microsatellite markers (SSR) associated with heat tolerance. The rice cultivars NERICA– L 44 (heat tolerant) and Uma (heat susceptible) were crossed to generate F^1^ and F^2^ populations. The F^2^ population was subjected to heat stress at >38°C and the 144 F^2^ plants were evaluated for their tolerance. The results note that the mean of the F^2^ population was influenced by the tolerant parent with regards to the traits of plant height, membrane stability index, photosynthetic rate, stomatal conductance, evapotranspiration rate, pollen viability, spikelet fertility and 1000 grain weight. Ten each of the extremely susceptible and tolerant plants were selected based on the spikelet fertility percentage. Their DNA was pooled into tolerant and susceptible bulks and Bulked Segregant Analysis (BSA) was carried out using 100 SSR markers to check for polymorphism. The survey revealed a polymorphism of 18% between the parents. RM337, RM10793, RM242, RM5749, RM6100, RM490, RM470, RM473, RM222 and RM556 are some of the prominent markers that were found to be polymorphic between the parents and the bulks. We performed gene annotation and enrichment analysis of identified polymorphic markers. Result revealed that the sequence specific site of that chromosome mostly enriched with biological processes like metabolic pathway, molecular mechanism, and subcellular function. Among that RM337 was newly reported marker for heat tolerance. Expression analysis of two genes corresponds to RM337 revealed that *LOP1* (LOC_Os08g01330) was linked to high temperature tolerance in rice. The results demonstrate that BSA using SSR markers is useful in identifying genomic regions that contribute to thermotolerance.

## Introduction

Research into the genetic mechanism of heat tolerance is becoming increasingly important for the utilization of heat-tolerant genes in the development of new rice varieties. Advances in rice genomics research and the completion of the rice genome sequence have made it possible to identify and precisely map several genes through linkage to DNA markers. By determining the allele of a DNA marker, plants that possess favorable genes or quantitative trait loci (QTLs) may be identified based on their genotype (Foolad and Sharma, 2004). Many stress resistance genes that are tightly linked to SNP, SSR, and STS markers are available (Das et al., 2017). Radha et al. (2023) reviewed the impact of high temperatures on rice yield and grain quality parameters and QTLs linked to high temperature tolerance in rice. Marker-assisted selection (MAS) can integrate these genes into breeding populations in combination with conventional breeding approaches.

Bulked segregant analysis (BSA) is a technique that is used to rapidly identify markers that are tightly linked to genes for a given phenotype (Zou et al., 2016). With the release of sequenced genomes, the combined application of bulked segregant analysis (BSA) and next-generation sequencing technology represents a new way to accelerate the identification of candidate genes controlling important agronomic traits in various crops (Tiwari et al., 2016). As heat-stress responses are governed by polygenes or QTL/thermotolerance genes, concerted efforts should be made to understand tolerance mechanisms at molecular and physiological levels.

Due to the low cost, wide availability, and easy technique, in addition to their high polymorphism rate, simple sequence repeats (SSR) are the most widely employed markers in MAS in the present times (Gao et al., 2016; Beena et al., 2018b; Rejeth et al., 2020). Due to parental genetic diversity, each offspring group of a cross is distinct at morphological and molecular levels. Even though many markers linked to different traits for resistance to heat stress are reported, the markers verified in a particular cross of parental genotypes may not be applicable to other populations. Therefore, using bulked segregant analysis, the current work sought to identify the molecular markers linked to high temperature tolerance in rice from the F_2_ population of NERICA-L 44 and Uma, and the identified markers were subjected to gene annotation and enrichment analysis.

## Materials and methods

### Location

The experiment was carried out at the College of Agriculture in Vellayani, Kerala. The institute is in southern India (8.44° N, 76.99° E), where the climate is typically humid and tropical, with average summer temperatures reaching around 35°C and average winter temperatures of about 20°C.

### Crop growth conditions

The experiment was conducted as a pot culture study in a control facility. The size of each pot was 25 × 15 cm, with soil and farmyard manure filled in a 2:1 ratio. The average weight of the pot after filling was about 6 kg. The seedlings were raised in a nursery and transplanted into the pots after 18 days. Fertilizer was applied as recommended (100:60:40 kg ha^−1^ of N:P_2_O_5_:K_2_O) for this region. The first dose was applied as a basal dressing in each pot before transplanting, and the remaining amount was applied as a top dressing 15 days after transplanting, at the maximum tillering and booting stage.

The F_2_ seeds produced from the F_1_ progeny were germinated and raised following standard crop management according to the package of practices (KAU [Kerala Agricultural University], 2016). The plants were grown in lowland conditions, i.e., with standing water for most of the crop duration except for mid-season drainage at 40 and 60 days. Until the maximum tillering stage, the plants were kept in normal environmental conditions with the average daytime temperature ranging around 30–32°C and night temperature around 24–26°C. The plants were thereafter moved to a high-temperature polyhouse, where the average temperature was around 38–42°C during the day. The plants were kept under high temperature conditions until their harvest. The physiological parameters were recorded at the flowering phase and yield parameters were recorded at the grain-filling phase.

### Materials used

The rice varieties Uma and NERICA L-44 (New Rice for Africa-Lowland 44) were selected for the present study. Uma (MO-16) is a popular and high-yielding rice variety developed by the Kerala Agricultural University, but it is susceptible to high temperature stress (Waghmare et al., 2018), while the rice NERICA L-44 (NL-44), derived from the crossing of the African rice (*O. glaberimma* Steud.) and the Asian rice (*O. sativa* L.), was reported as a heat-tolerant line (Bahuguna et al., 2015; Ravikiran et al., 2020). The two rice varieties, Uma and NL-44 were sown in a staggered planting pattern to coincide with the date of flowering between the two parents. Standard crop management was followed according to the package of practices recommend for this region (KAU [Kerala Agricultural University], 2016), at ambient temperature of 26–34°C without any stress during the crop growth period. The variety NL-44 (donor) was crossed with Uma to produce a good number of F_1_ seeds. The F_1_ seeds were raise in the field to produce 150 F_2_ seeds.

### Morphological parameters recorded

The F_2_ lines were evaluated along with their two parents, NL-44 and Uma, by calculating the mean and the deviation of each plant from the mean. The range of data was estimated by dividing the data into quartiles, and the shape of the normal curve for each parameter was determined. The physiological parameters were recorded at the flowering phase, and yield parameters were recorded at the grain filling phase.

Morphological parameters were recorded on plant height (cm), tiller number, number of productive tillers, days to 50% flowering, time of anthesis, panicle length (cm), 1,000 grain weight (TGW) (g), spikelet fertility (SF) (%) was calculated using the formula: SF (%) = (Number of fertile spikelets/Total number of spikelets) × 100.

The physiological parameters pollen stainability (%), cell membrane stability, photosynthetic rate, transpiration rate, stomatal conductance, and leaf temperature were measured. Pollen stainability (%) (which indicates pollen viability) was calculated by the iodine-potassium iodide method. The pollen viability percentage was calculated by the formula: (number of stained pollen grains/total number of pollen grains) × 100. Cell membrane stability was measured according to the procedure given by Sairam et al. (1997). The membrane stability index (MSI) was calculated using the formula: MSI = [1 − (C1/C2)] × 100, where C1 and C2 are the initial and final electrical conductance measured when leaf discs (100 mg) were heated in a water bath at 40°C (30 min) and 100°C (10 min), respectively. The photosynthetic rate, transpiration rate, stomatal conductance, and leaf temperature were measured using an Infra-Red Gas Analyzer (LI-COR 6400XT, USA).

### Genomic DNA isolation and marker assay

The DNA was isolated using the method suggested by Dellaporta et al. (1983), and the quality and quantity were estimated using the ratio of absorbance at 260 nm and 280 nm. A ratio (A_260_/A_280_) of 1.8–2.0 was obtained for the samples, which is good quality DNA. The PCR reaction to check for DNA polymorphism was carried out by preparing a standard mixture having the following components: 10× Taq buffer (2 µl), forward primer (0.75 µl), reverse primer (0.75 µl), dNTP mix (1.5 µl), Taq polymerase (1 µl/5 reactions), and sterile water (12.7 µl), and DNA template (1 µl/reaction). The thermal profile of the PCR cycling conditions was standard except for the annealing temperature, which was specific to the individual markers. The cycling conditions for the PCR reaction were: 1) initial denaturation (95°C—5 min), 2) denaturation (94°C—1 min), 3) annealing (57°C—1 min), 4) extension (72°C—2 min), and 5) final extension (72°C—5 min). Steps 2 to 4 were repeated for 30 cycles. The quality of the PCR products was checked by separating them on a 3% agarose gel using the electrophoresis technique.

### Identification of genetic loci linked with heat tolerance traits through BSA

Bulked segregant analysis (BSA) was utilized to identify polymorphic markers between the tolerant and the susceptible bulks. The markers that differentiated between the tolerant and susceptible bulks through the difference in the amplicon size of the marker, separated through gel electrophoresis, were considered polymorphic. Through BSA, the polymorphic markers between the tolerant and susceptible bulks were used to study the segregation of the alleles in the individual lines constituting the tolerant and susceptible bulks.

In this method, based upon the phenotypic evaluation of the F_2_ lines using spikelet fertility percentage as a phenotypic marker for heat tolerance, 10 extremely tolerant and 10 extremely susceptible lines were selected. DNA was extracted from the selected 10 heat-tolerant and 10 susceptible segregant lines, and an equal quantity of DNA was pooled. The bulked DNA samples were screened using 100 simple sequence repeat (SSR) primers. The putative polymorphic markers between the bulks were checked among the parents as well as the individual lines constituting the tolerant and susceptible bulks. Polymorphic markers were subjected to gene annotation and enrichment analysis using all the genes separately (http://bis.zju.edu.cn/ricenetdb/).

### Gene expression

The leaf samples were taken at the vegetative stage. The total RNA was isolated using TRI reagent from HiMedia. The isolated samples were quantified using a Qubit reagent. The cDNA was prepared using RT superscript enzyme as well as without RT superscript enzyme. The genes *LOP1* (LOC_Os08g01330) and *LOP2* **(**LOC_Os08g0112) were amplified using primers of concentration 10 picomoles per microliter using a cDNA quantity corresponding to 10ng of total RNA used for cDNA preparation. The qPCR was carried out using a 2× master mix containing SYBR Green reagent. For each sample, three replicates were used. In the analysis software, samples and controls were marked. After the completion of qPCR, the data was auto analyzed using CFX Analysis Manager. The primer sequence is given in **Table 1**.

**Table 1:** List of primers used for qRT-PCR.

Gene name	Forward primer	Reverse primer
**LOC_Os08g01330 (*LOP1*)**	CGACTGGAACCTGCTCAC	CGATGAAGCCTGACGAAGAA
**LOC_Os08g01120 (*LOP2*)**	TACGCTACGAGCAGGACTT	CGTTGACGAGCACGATGA

## Results

### Phenotypic evaluation of F_2_ population of NL-44 × Uma for high temperature tolerance

#### Growth parameters

**Figure 1:**
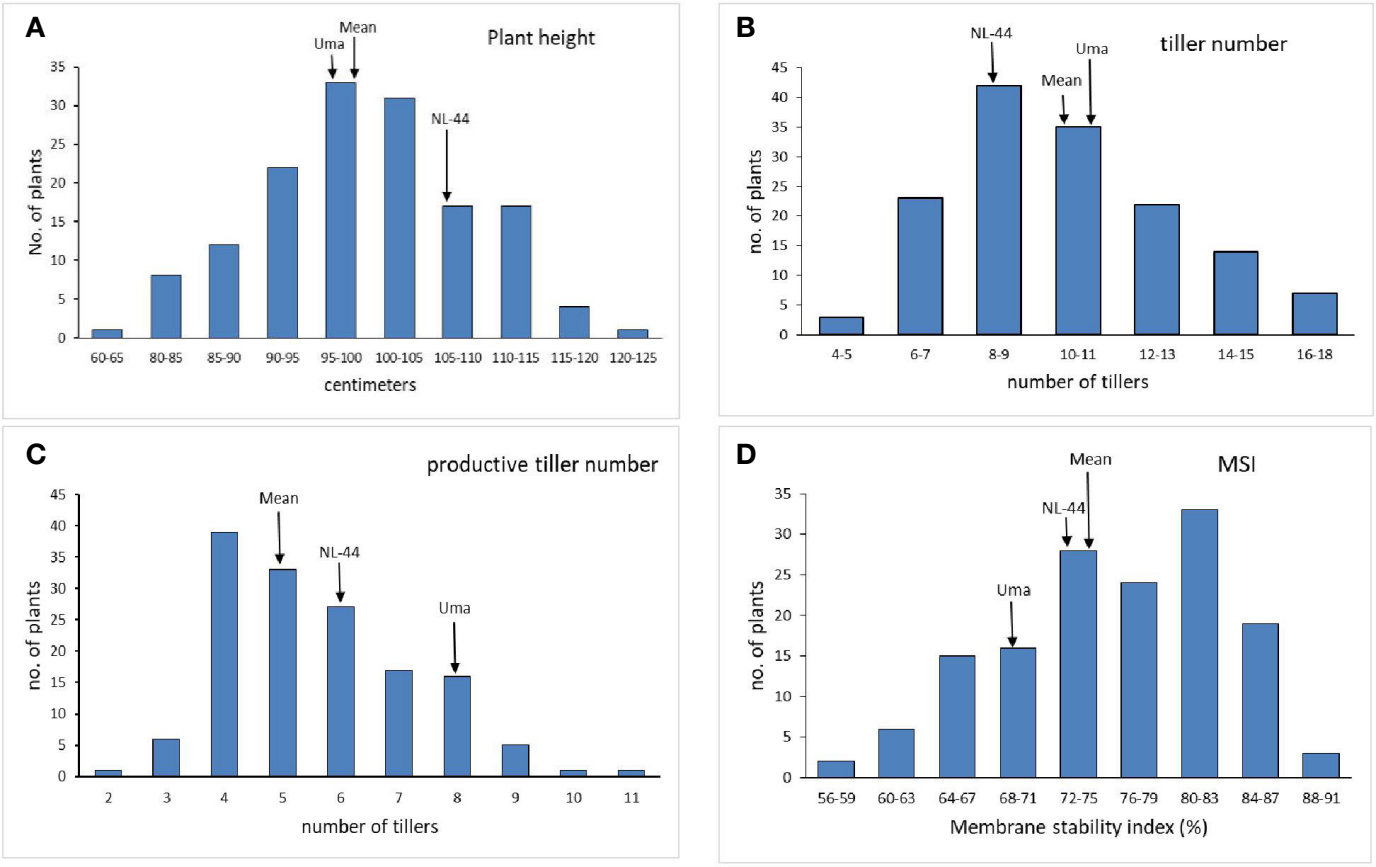
Frequency distribution of plant height **(A)**, tiller number **(B)**, productive tiller number **(C)**, and membrane stability index (MSI) **(D)** under the effect of high temperature stress among the F_2_ population.

The mean plant height (**Figure 1**) of the population was 99.09 cm, with the minimum height being 64 cm and the maximum recorded at 121 cm. The plant height of the Uma variety was recorded at 96.73 cm, while NL-44 recorded 105.62 cm. The average number of tillers was 10.17, with the highest number recorded as 17 and the least being 5 (**Table 2**). The number of tillers recorded in Uma was 11, while NL-44 produced eight. The average number of productive tillers was 5.58, with the lowest number being 2, while the maximum productive tiller number was 11. The Uma variety recorded eight productive tillers under heat stress, whereas NL-44 recorded six tillers. The mean MSI of the population was calculated at 75.84%, with a minimum MSI of 56% and a maximum MSI of 90%. The MSI of the Uma variety was 68% while that of NL-44 was 79%.

**Table 2:** Descriptive statistics of various parameters of the F_2_ population of NL-44 × Uma under the effect of heat stress.

Descriptive statistical parameter	Plant height (cm)	tiller number	Productive tiller number	Days to flowering	Time of anthesis (a.m.)	MSI (%)	Photosynthetic rate (µmol cm^−2^s^−1)^	Stomatal conductance (mol m^−2^s^−1)^	Transpiration rate (mmol m^−2^s^−1)^	Leaf temperature (˚C)	Pollen viability (%)	Panicle length (cm)	Spikelet fertility (%)	1,000 grain weight (g)
Mean	99.09	10.17	5.58	87.5	9:47	75.84	24.35	0.23	5.91	34.41	77.76	22.94	41.54	22.11
Standard Error	0.74	0.23	0.13	0.53	0.06	0.60	0.28	0.0029	0.051	0.05	0.56	0.17	1.05	0.14
Median	99	10	5	87	9:47	76.5	25.3	0.23	5.82	34.43	79	23.1	40.90	22.5
Mode	104	9	4	91	10:00	81	23.4	0.234	5.14	34.91	81	24.2	46.53	22.5
Standard Deviation	9.01	2.83	1.66	6.41	0.75	7.30	3.47	0.035	0.62	0.60	6.82	2.07	12.69	1.74
Sample Variance	81.20	8.03	2.76	41.20	0.56	53.38	12.08	0.0012	0.39	0.36	46.53	4.31	161.07	3.053
Kurtosis	0.66	−0.34	−0.05	−0.64	−0.52	−0.58	−0.51	0.37	−0.86	−0.74	0.035	−0.32	−0.05	−0.76
Skewness	−0.31	0.44	0.61	0.01	0.08	−0.40	−0.64	0.79	0.16	−0.15	−0.62	−0.18	0.33	−0.58
Range	57	12	9	30	3:45	34	14.1	0.164	2.52	2.67	32	10.2	60.1	6.6
Minimum	64	5	2	71	8:00	56	15.6	0.181	4.63	33.14	59	17.6	15.17	18.2
Maximum	121	17	11	101	11:45	90	29.7	0.345	7.15	35.81	91	27.8	75.27	24.8
Count	146	146	146	146	146	146	146	146	146	146	146	146	146	146
Confidence Level (95.0%)	1.47	0.46	0.27	1.04	0.12	1.19	0.56	0.0058	0.10	0.098	1.11	0.33	2.075	0.28

#### Characteristics of flowering

**Figure 2:**
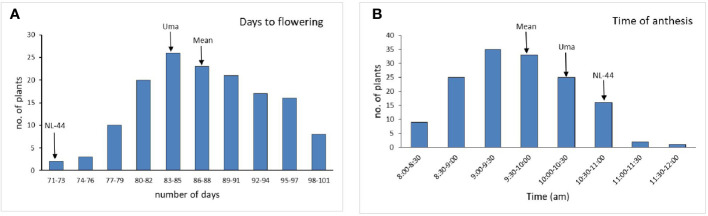
Frequency distribution of days to flowering **(A)** and time of anthesis **(B)** under the effect of high temperature stress among the F_2_ population.

The population’s mean flowering time was calculated to be 87.5 days, with a range of 30 days, with the least being 71 days and the most being 101 days. The variety Uma was recorded to flower in around 83 days, while NL-44 took 71 days to flower. The mean time of anthesis (**Figure 2**) for the F_2_ population was 9:47 am, with a range of 3 h and 45 min, with the earliest anthesis occurring at 8:00 am and the most delayed anthesis occurring at 11:45 am. The time of anthesis of the Uma variety was recorded at 10:15 am, while that of NL-44 was 10:45 am. The population’s mean pollen viability percentage was calculated to be 77.76%. This was within a range of 32%, with the lowest pollen viability percentage being 59% and the highest pollen viability percentage being 91%. The pollen viability of the Uma variety was 74%, while NL-44 was 86.67%.

#### Gas exchange related parameters

**Figure 3:**
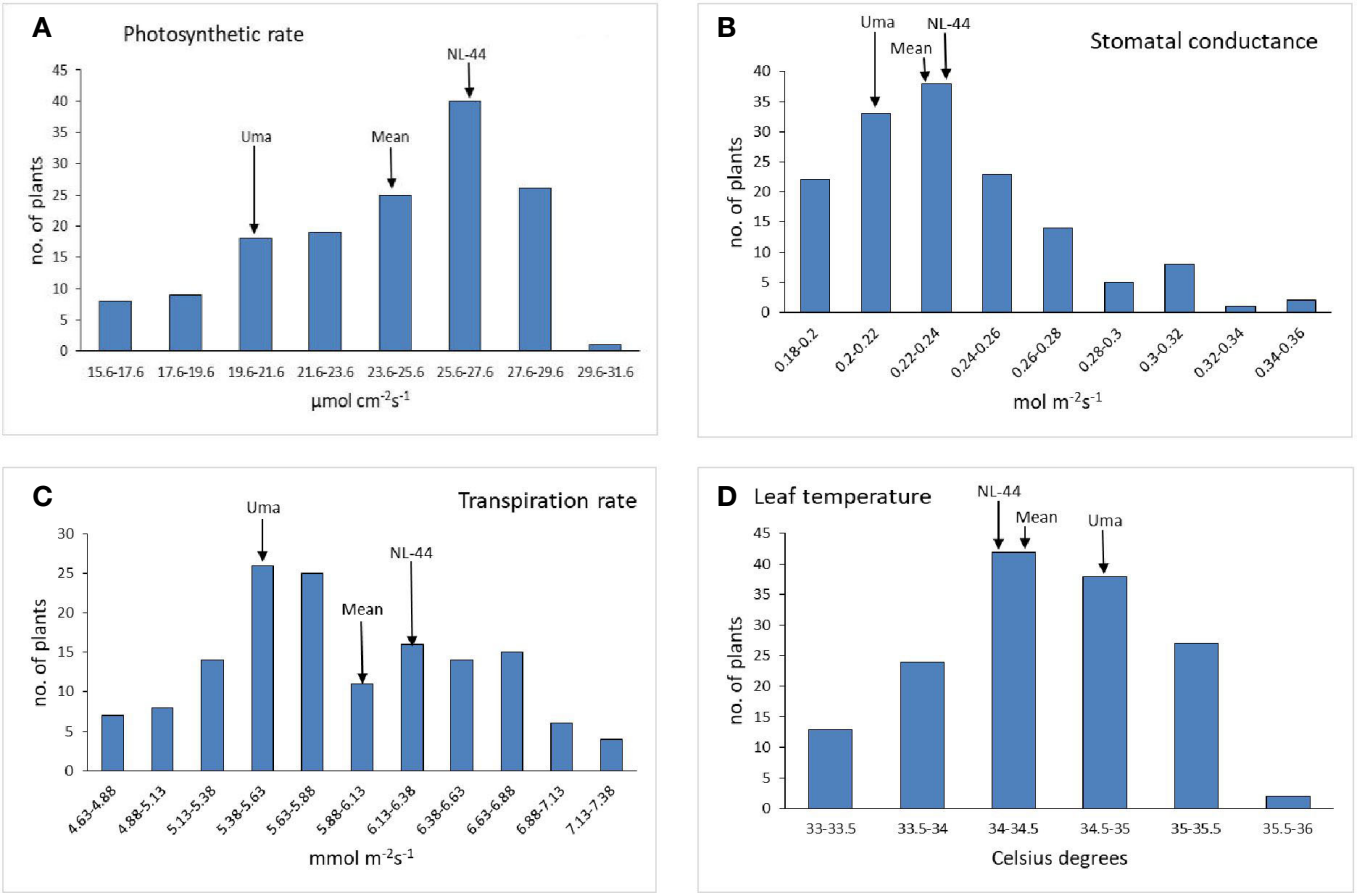
Frequency distribution of photosynthetic rate **(A)**, stomatal conductance **(B)**, transpiration rate **(C)**, and leaf temperature **(D)** under the effect of high temperature stress among the F_2_ population.

The population’s mean photosynthetic rate (Pn) was calculated to be 24.35 mol cm^−2^s^−1^, with a minimum Pn of 15.6 mol cm^−2^s^−1^ and a maximum Pn of 29.7 mol cm^−2^s^−1^. The Pn of the Uma variety was 20.68 µmol cm^−2^s^−1,^ while that of NL-44 was 27.21 µmol cm^−2^s^−1^. The mean stomatal conductance of the F_2_ population was 0.234 mol m^−2^s^−1^, where the maximum Gs was recorded at 0.345 mol m^−2^s^−1^ and the minimum Gs was 0.181 mol m^−2^s^−1^. The Gs of the Uma variety was recorded to be 0.213 mol m^−2^s^−1^, while that of NL-44 was 0.236 mol m^−2^s^−1^. The mean transpiration rate of the F_2_ population was 5.91 mmol m^−2^s^−1^ with a range of 2.52 mmol m^−2^s^−1^, where the maximum transpiration rate was recorded as 7.15 mmol m^−2^s^−1^ and the minimum was 4.63 mmol m^−2^s^−1^. The transpiration rate of the Uma variety was recorded at 5.45 mmol m^−2^s^−1^, while that of NL-44 was 6.24 mmol m^−2^s^−1^. The average leaf temperature was calculated to be 34.4°C, with a maximum of 35.8°C and a minimum of 33.1°C. The leaf temperature of the Uma variety was 34.87°C, and that of the NL-44 variety was 34.16°C (**Figure 3**).

#### Yield parameters

**Figure 4:**
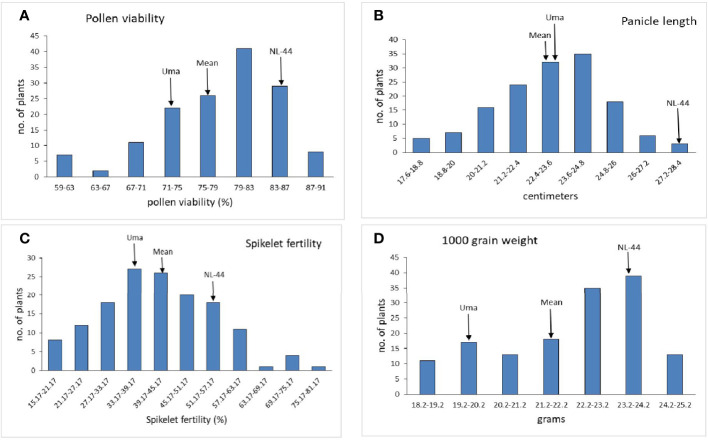
Frequency distribution of pollen viability **(A)**, panicle length **(B)**, spikelet fertility **(C)**, and 1,000 grain weight **(D)** under the effect of high temperature stress among the F_2_ population.

The mean panicle length of the population (**Figure 4**) was calculated as 22.94 cm, having a range of 10.2 cm, with the least panicle length of 17.6 cm and the highest panicle length of 27.8 cm. The panicle length of the Uma variety was recorded at 23.4 cm, and that of NL-44 was 27.62 cm. The mean spikelet fertility percentage of the population was 41.54% with the minimum spikelet fertility of 15.17% and the maximum recorded as 75.27%. The spikelet fertility of the Uma variety was calculated as 35.16% while that of NL-44 was 56.15%. The mean thousand grain weight (TGW) of the population was 22.11 g, with the least TGW of 18.2 g and the maximum recorded at 24.8 g. The TGW of the Uma variety was recorded at 20.14 g, while that of NL-44 was 23.27 g.

#### Correlation analysis

The correlation matrix of the parameters assessed in the F_2_ population under high temperature stress is presented in **Table 3**. The tiller number was positively correlated with the productive tiller number, days to flower, and pollen viability. Similarly, the productive tiller number was positively correlated with the membrane stability index. The membrane stability index was significantly correlated with pollen viability, spikelet fertility, and 1,000 grain weights in a positive manner. Pollen viability was also found to have a significant positive correlation with spikelet fertility and 1,000 seed weights. The correlation between spikelet fertility and 1,000 grain weights was positive at the p ≤0.001 level. The parameters of photosynthetic rate, evapotranspiration rate, stomatal conductance, and leaf temperature were positively and strongly correlated with each other. The time of anthesis was negatively correlated with spikelet fertility and 1,000 grain weight, while pollen viability was negatively correlated with leaf temperature.

**Table 3:** Correlation matrix of F_2_ population under high temperature stress.

	Plant Height	No. of tillers	Productive tiller no.	Days to flowering	Time of anthesis	MSI	Pollen Viability	Panicle length (cm)	Spikelet fertility	1000 seed weight	Pn	E	Gs	T
Plant Height	1	0.073	0.157	−0.014	0.023	−0.002	−0.074	0.095	0.022	−0.069	0.006	−0.039	0.054	0.041
No. of tillers	0.073	1	0.789***	0.195*	0.151	0.136	0.171*	0.035	0.079	0.071	−0.197*	−0.15	−0.151	−0.246**
Productive tiller no.	0.157	0.789***	1	0.017	0.102	0.177*	0.161	0.002	0.115	0.112	−0.209*	−0.178*	−0.139	−0.261**
Days to flowering	−0.014	0.195*	0.017	1	0.023	0.133	−0.007	−0.001	−0.004	0.041	0.096	0.061	0.133	0.035
Time of anthesis	0.023	0.151	0.102	0.023	1	−0.111	−0.151	−0.009	−0.165*	−0.184*	0.022	0.047	0.051	0.007
MSI	−0.002	0.136	0.177*	0.133	−0.111	1	0.458***	0.06	0.324***	0.274***	0.039	−0.008	0.035	−0.073
Pollen Viability	−0.074	0.171*	0.161	−0.007	−0.151	0.458***	1	0.074	0.315***	0.298***	−0.116	−0.161	−0.158	−0.253**
Panicle length (cm)	0.095	0.035	0.002	−0.001	-0.009	0.06	0.074	1	0.001	−0.052	−0.144	−0.165*	−0.037	−0.226**
Spikelet fertility	0.022	0.079	0.115	−0.004	−0.165*	0.324***	0.315***	0.001	1	0.861***	0.036	−0.015	−0.055	−0.107
1,000 seed weight	−0.069	0.071	0.112	0.041	−0.184*	0.274***	0.298***	−0.052	0.861***	1	−0.026	−0.064	−0.115	−0.151
Pn	0.006	−0.197*	−0.209*	0.096	0.022	0.039	−0.116	−0.144	0.036	−0.026	1	0.896***	0.679***	0.764***
E	−0.039	-0.15	−0.178*	0.061	0.047	−0.008	−0.161	−0.165*	−0.015	−0.064	0.896***	1	0.706***	0.835***
Gs	0.054	−0.151	−0.139	0.133	0.051	0.035	−0.158	−0.037	−0.055	−0.115	0.679***	0.706***	1	0.66***
T	0.041	−0.246**	−0.261**	0.035	0.007	−0.073	−0.253**	−0.226**	−0.107	−0.151	0.764***	0.835***	0.66***	1

***indicates significance at 0.001 level (two tailed), ** indicates significance at 0.01 level (two tailed) and * indicates significance at 0.05 level (two tailed).

### Identification of polymorphic molecular markers linked to high temperature tolerance

**Figure 5:**
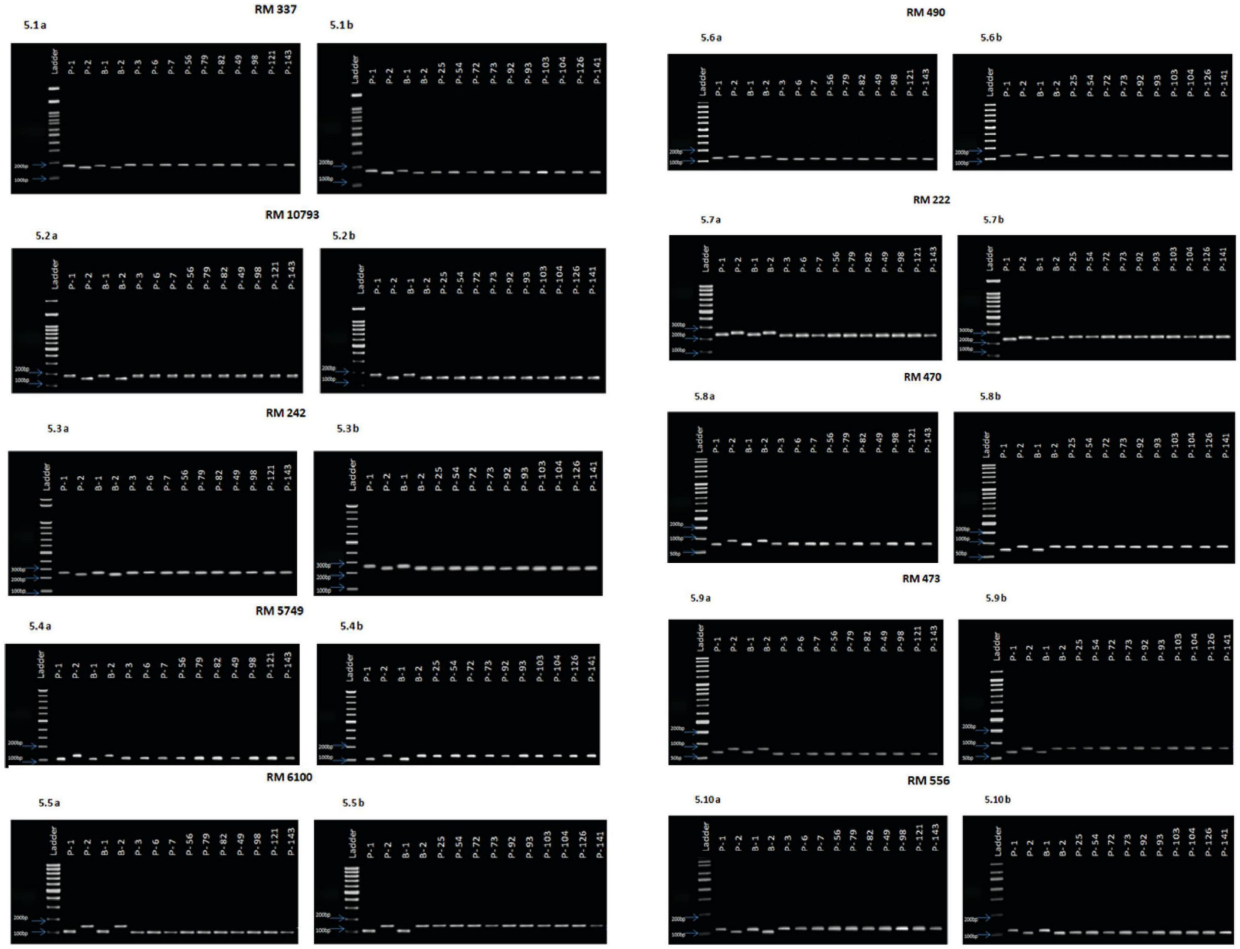
**(5.1A).** Amplification pattern of SSR markers 100 bp Ladder, P1—NL 44, P2—Uma, B1—Tolerant bulk, B2—Susceptible bulk, P 3, P 6, P 7, P 56, P 79, P 82, P 49, P 98, P 121, P 143—Tolerant lines. **(5.1B)**. Amplification pattern of SSR markers100 bp Ladder, P1—NL 44, P2—Uma, B1—Tolerant bulk, B2—Susceptible bulk, P 25, P 54, P 72, P 73, P 92, P 93, P 103, P 104, P 126, P 141—Susceptible lines,.

Based on the results of the phenotypic evaluation of the 144 F_2_ generation plants, 10 plants from extremely heat tolerant and extremely heat susceptible were selected. The F_2_ generation plant numbers P3, P6, P7, P56, P79, P82, P49, P98, P121, and P143 were in the extremely tolerant group, and plant numbers P25, P54, P72, P73, P92, P93, P103, P104, P126, and P141 were in the extremely susceptible group. Based on the amplification pattern obtained, 18 markers were determined to exhibit polymorphism between the parents (**Table 4**). The amplification pattern and polymorphism level between the parents, bulks, and debulks of 10 markers (RM337, RM10793, RM242, RM5749, RM6100, RM490, RM3475, RM470, RM473, and RM556) clearly indicate their ability to separate the heat tolerance or susceptible plants (**Figure 5**).

**Table 4:** List of SSR markers exhibiting polymorphism between parents.

Marker	Chromosome
RM237	1
RM490	1
RM3475	1
RM10793	1
RM3586	3
RM554	3
RM471	4
RM470	4
RM5749	4
RM413	5
RM320	7
RM473	7
RM337	8
RM556	8
RM310	8
RM242	9
RM6100	10
RM222	10

### Gene annotation and enrichment analysis

To get a deeper insight into how these genes participate in heat tolerance, we performed GO (gene annotation) and enrichment analysis (http://bis.zju.edu.cn/ricenetdb/) on all the genes separately (**Figure 5**). Result revealed that the sequence-specific site of that chromosome was mostly enriched with biological processes like metabolic pathways, molecular mechanisms, and subcellular function. Out of the 10 markers identified in the BSA (RM337, RM10793, RM242, RM5749, RM6100, RM490, RM3475, RM470, RM473, and RM556), nine of the markers have been previously reported for heat tolerance traits, while one marker, RM337, is newly identified in the present study.

The genes in the 200 kb vicinity of the RM markers were retrieved from the Rice Annotation Project database (https://rapdb.dna.affrc.go.jp/viewer/gbrowse/irgsp1/, accessed on 18 August 2022) based on their putative function related to heat tolerance. Upon screening of the loci in the proposed region, known heat tolerance genes and characterized genes were identified (**Supplementary Table 1**: Gene annotation and enrichment analysis).

Annotation, GO enrichment analysis, and trait ontology (TO) were performed for all the significant markers. The marker information was searched through the RiceNetDB and Oryzabase databases. RM 490, which is located at chromosome number 01, had 426 genes that were significantly enriched and involved in different pathways, among them 10 that were involved in heat tolerance. Four of them were characterized genes (*OsHSP16.9C* (Jung et al., 2014), *OsHsp18.0* (Kuang et al., 2017), *WRKY10* (Chen et al., 2022), and *OsDREB2A* (TO:0000259)) and have an active role in abiotic stress tolerance and heat shock, and are involved in seed maturation, anther traits, leaf senescence, seed germination, plant growth and development, signal transduction, and metabolic processes. RM 10793, located on chromosome number 01, showed 788 genes significantly enriched and involved in different pathways (SF1). A total of 19 genes were known for heat tolerance, including 10 characterized genes (*OsHSP16.9C*) (Jung et al., 2014), *WRKY10* (Chen et al., 2022)*, OsHsp18.0* (Kuang et al., 2017), *OsNTL3* (Liu et al., 2020), *OsDREB2AOsNLP3*, *OsSUN2*, *OsGAS2*, *OsTGA5*, and *OsGI with* TO:0000259), which have active roles in abiotic stress tolerance, osmotic stress, heat tolerance, seed maturation, chlorophyll content, and flowering time regulator. The RM 470, located on chromosome number 4, showed 880 genes significantly enriched and involved in different pathways. Approximately, 17 of them were known for heat tolerance-related activities, including eight characterized genes for different traits (*BAD1*, *OsABA1*, *OsZEP*, *OsFPFL4*, *OsNLP2*, *OsWRKY36* with TO:0000259), *OsFKBP65* (Magiri et al., 2006), and *RPL6* (Moin et al., 2016), which have an active role in temperature tolerance, abiotic stress tolerance, and modulating root and flower development. The marker RM 5749, located on chromosome number 04, showed 298 genes significantly enriched and involved in different pathways (SF2). Five were known for heat tolerance, including two characterized genes (*OsFKBP65* (Suzuki et al., 2015) and *OsFPFL4* (TO:0000259)) which have an active role in tissue-specific heat tolerance, abiotic stress tolerance, pollen, root, and anther development. Marker RM 473, located on chromosome number 07, showed 922 genes significantly enriched and involved in a different pathway (SF3). Five were known for heat tolerance, including one characterized gene (*ZFP177*), which actively contributes to temperature stress tolerance (Huang et al., 2008). The annotation of the marker RM 337 region on chromosome number 8 showed 23 genes significantly enriched and involved in different pathways, out of which seven were known characterized genes controlling traits like chlorophyll content, plant growth hormone sensitivity, inflorescence development trait, bacterial blight disease resistance, UV light sensitivity, seed development trait, and drought tolerance. Among these genes *NAC31* (*OsSWN3*; *LOC_Os08g01330*) a NAC involved in regulation of cellulose synthesis, regulation of secondary wall biosynthesis (*Os08t0103900-01*) said to be involved in heat tolerance. NAC transcription factors are also responsive to thermomemory in Arabidopsis (Alshareef et al., 2022). This marker is position centric between these two genes. LOC_*Os08g01120* (86,388–87,854) RM 337 (152,299–152,485) LOC_*Os08g01330* (210,422–212,411).

**Figure 6:**
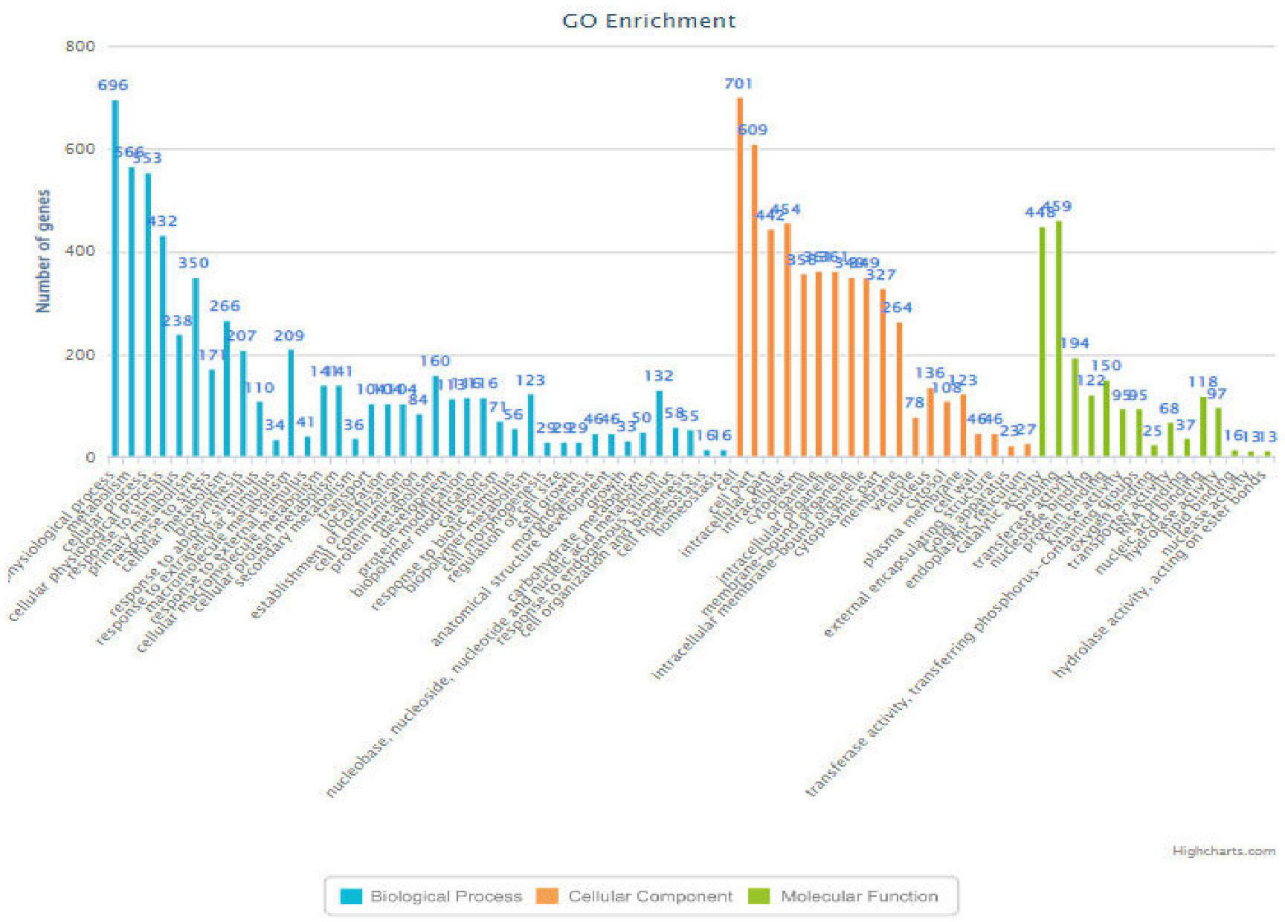
Bar-chart (RM6100) indicating the functions of the genes associated with various biological processes along with their frequency.

The RM 242, located on chromosome number 09, had 489 genes significantly enriched and involved in different pathways, among them eight were known for heat tolerance and included six characterized genes (*ACO1* (TO:0000259), *OsFBN1* (Li et al., 2019), *OsHTAS* (Jan et al., 2021), *OsNSUN2* (Tang et al., 2020), and *Amy3C* and *PDIL2;3* (TO:0000259)) that have an active role in heat tolerance at the seedling stage grain-filling percent and RNA methyltransferase of *OsNSUN2* for heat tolerance. Similarly, RM 6100, located on chromosome number 10, showed 246 genes significantly enriched and involved in different pathways (**Figure 6**). Seven were known for heat tolerance, including one characterized gene (*CYP75B3/F3’H;* Trait ontology—0000259) in cereal grasses that belongs to the cytochrome P450 family and catalyzes the 3′-hydroxylation of the B-ring of flavonoids (Jia et al., 2019). The RM 222, on chromosome number 10, was annotated and showed 170 genes significantly enriched and involved in different pathways, out of which 14 were known characterized genes controlling traits like growth and development trait, bacterial leaf streak disease resistance, grain size, grain weight, leaf development trait, plant height, cold tolerance, salt tolerance, days to head, drought tolerance, brown planthopper resistance, anther color, pollen fertility, anther shape, anther length, etc. Among these genes *OsADF* (*LOC_Os10g03660*, anther development F-box), involved in anther color, pollen fertility, anther shape, and anther length traits and was selected for heat tolerance gene. *OsADF* gene family participate in plant abiotic stress response or tolerance (https://thericejournal.springeropen.com/articles/10.1186/1939-8433-5-33).

### Gene expression analysis

**Figure 7:**
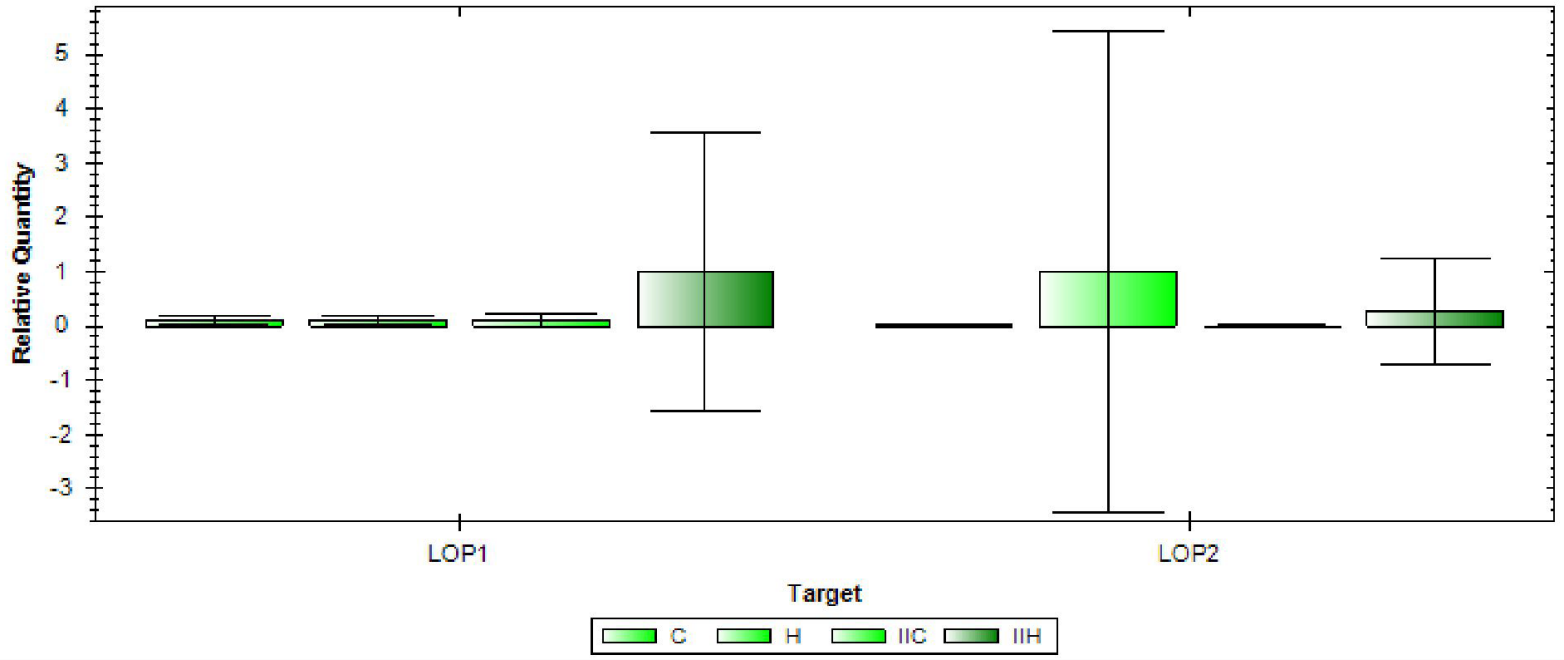
Gene expression of LOP1 and LOP2 [(C—Uma (control), H—Uma (high temperature), IIC-NL-44 (control), IIH-NL-44 (high temperature)].

The genes *LOP1* (LOC_Os08g01330) and *LOP2* **(**LOC_Os08g0112) were found to be associated with the marker RM337, and therefore their expression was analyzed in the two rice varieties under both control and high temperature conditions (**Figure 7**). *LOP1* was found to be significantly upregulated in the NL-44 variety under high temperature condition compared to the normal temperature conditions and susceptible variety, Uma. *LOP1* is a NAC transcription factor that is reported to be involved in the regulation of cellulose synthesis, secondary wall biosynthesis (*Os08t0103900-01*), and inflorescence development, all of which contribute to better physical tolerance characteristics during vegetative growth as well as the maintenance of an improved pollination rate, which can be observed in the tolerant NL-44. On the other hand, the gene *LOP2* was found to be upregulated in both varieties under the higher temperature condition compared to their respective controls. However, the relative expression in Uma was higher than in NL-44. *LOP2*, also known as *OsMOT1* (Cobb et al., 2021), is a molybdate transporter that is involved in the uptake and translocation of molybdate (*Os08t0101500-01*). This gene is reported to be involved in drought, cold, and salt tolerance, as well as seed development. Its higher relative expression in the heat-susceptible variety Uma might indicate that it might possess tolerance traits for other traits other than high temperature as abiotic stresses are found to be affecting the crops in conjunction. Comparatively, the upregulation in NL-44 might be attributed to its improved tolerance under heat stress conditions.

## Discussion

The effects of climate change, which include rising temperatures, longer droughts, and desertification, are anticipated to make several places unfit for agricultural use. Rice has been produced under a variety of climatic conditions throughout history; high temperatures are particularly detrimental, causing spikelet sterility which lowers rice production even when other yield components develop normally. Long-term rice yield in tropical, subtropical, and temperate regions is imperiled by heat stress during flowering (Beena, 2013). Therefore, it is imperative that heat-tolerant rice cultivars be created for future rice production (Costa and Farrant, 2019). According to short-term forecasts, the rice output in South Asia might fall by 10% of the yield by 2030. (Lobell et al., 2008). According to the medium- to long-term projections of Cline (2008), rice yields in developing countries might decline by 10%–25% by 2080, and in India, by 30%–40%. Since heat tolerance is quantitative in nature, the underlying genetic process is complicated. Rarely are these prospective genes used to develop high-yielding, heat-resistant rice cultivars. Therefore, it is essential to conduct more studies to uncover probable genes and linked markers for heat tolerance.

### Evaluation of F_2_ population

#### Influence of high temperature stress on growth

The Uma variety recorded a lower height compared to NL-44, with the mean of the population lying between the two genotypes. Kilasi et al. (2018) identified several QTLs that were associated with shoot length and root length under high temperature conditions. NL-44 recorded lower tiller number compared to Uma, with the mean lying in between with 10 tillers. The mean tiller number was found to be greater than in NL-44 variety and closer to the Uma variety. The mean of the population was lower than both the genotypes with NL-44 producing lower number compared to that of Uma.

Comparing the productive tiller number, the lower value of the mean shows that the trait in the F_2_ population was influenced to a greater extent by NL-44 and was depressed compared to both parents. Regarding membrane stability index, the mean of the population was greater than both parental genotypes, clearly indicating that most of the population has the potential for producing MSI greater than that of the parents. A similar trend was observed in the study by Prince et al. (2015) in the F_8_ population of IR20 and Nootripathu under drought conditions.

#### Influence of high temperature stress on characteristics of flowering

The genotype NL-44 took the least number of days to attain flowering compared to that of Uma. The mean value being greater than Uma shows that the trait in most plants has been influenced by Uma. The variety NL-44 had a greater pollen viability percentage compared to Uma with the population mean value greater than the susceptible variety Uma. This indicates that the majority of the F_2_ population has a pollen viability percentage that has been influenced by the tolerant parent, NL-44.

The time of anthesis for the F_2_ population had a range spanning 3 h and 45 min, where the mean of the population was lower than that of both parents. This shows that most of the population had an earlier time of onset. Among the parents, Uma had earlier anthesis compared to NL-44. The earlier time of anthesis is a trait that is useful for escaping the high temperature stress. Bheemanahalli et al. (2017) reported a near-isogenic line (IR64 + qEMF3) possessing the early morning flowering (EMF) trait to have minimized spikelet sterility by 71%. The beneficial effects of the novel QTL, qEMF3, on mitigating heat stress damage in rice at flowering were also observed by Hirabayashi et al. (2015). Therefore, incorporating such traits could be a useful strategy to combat heat stress.

#### Influence of high temperature stress on gas exchange related parameters

The Uma variety recorded a lower photosynthetic rate compared to NL-44, with the mean of the population lying between the two genotypes. The mean of the population being greater than the susceptible Uma shows that most of the population had a higher photosynthetic rate, which was influenced by the tolerant NL-44. The Uma variety recorded a lower stomatal conductance compared to NL-44, with the mean lying between the parents. Similar results have also been noted under high temperature conditions, wherein the lower photosynthetic rate and stomatal conductance were observed in the susceptible rice varieties investigated (Beena et al., 2018a).

The Uma variety recorded a lower transpiration rate compared to NL-44, with the mean of the population lying between the two. The mean value being greater than that of the susceptible Uma variety indicates that most of the population had a higher transpiration rate. The variety NL-44 recorded a lower leaf temperature compared to Uma, with the mean lying between them. The mean being closer to NL-44 and lower than Uma shows that most of the population has a lower leaf temperature. The study by Stephen et al. (2022) also reported the decrease in canopy temperature of NL-44 under high temperature conditions.

#### Influence of high temperature on yield

The panicle length of the tolerant NL-44 was extremely high, while that of Uma was lower. The mean of the F_2_ population was lower than both the parental genotypes and relatively close to Uma. This clearly indicates that the panicle length of most of the population was influenced by the Uma variety. Zhu et al. (2017) explored the genetic basis of heat tolerance in rice and identified the QTL *qHTB3-3* on the third chromosome that was associated with heat stress at the booting stage. The tolerant parent NL-44 recorded a higher 1,000 grain weight compared to the susceptible parent, while the mean was greater than the Uma variety and in between the two parental genotypes. The mean being closer to NL-44 clearly indicates that the trait has been greatly influenced by the tolerant parent. Chen et al. (2021) indicated that the gene *OsSAP5* (stress-associated proteins) might be induced by spermidine, which prevents the damage to rice seed resulting from high temperature stress during the grain-filling stage. This would be beneficial for maintaining crop yield under stressful conditions.

The tolerant variety NL-44 had much higher spikelet fertility compared to its susceptible parent. The mean of the population lies between the two parental genotypes. The greater value of the mean compared to Uma indicates that this important trait for heat tolerance has been beneficially transferred from the parent NL-44 to most of the population. Takai et al. (2020), while studying QTLs associated with spikelet fertility noted that the expression of QTLs may be dependent on their genetic background. Therefore, the varieties selected and the alleles present in them must be carefully considered while undertaking breeding programs. The reduction in spikelet fertility in rice at higher temperatures was also corroborated by Beena et al. (2021) and Pravallika et al. (2020). Ye et al. (2022) used the strategy of marker-assisted pyramiding of “early morning flowering” and heat tolerance QTLs (qEMF3 and qHTSF 4.1) in rice, to ensure a higher spikelet fertility rate and enhance resilience to heat stress.

#### Analysis of correlation between the parameters

The 1,000-grain weight was significantly correlated with the tolerance contributing traits such as membrane stability index, pollen viability, and spikelet fertility which explains their positive impact on the yield characteristics. The positive correlation of the membrane stability index with pollen viability and spikelet fertility is a major contribution to the heat tolerance of the plants. The negative correlation of the time of anthesis with spikelet fertility and 1,000 grain weight indicate that an earlier time of anthesis is a trait that is beneficial in avoiding the effects of high temperature stress, leading to a higher grain yield. This observation of rice was also confirmed by Raghunath and Beena (2021).

The positive correlation of the stomatal conductance with the photosynthetic rate is important as a higher rate of gaseous exchange significantly improves the photosynthetic rate due to a greater influx of carbon dioxide, whereas the positive correlation between the photosynthetic rate and the evapo-transpiration points to the beneficial effect of transpirational cooling under high temperature stress (Vijayakumar and Beena, 2020). The negative correlation between leaf temperature and pollen viability shows that lower temperatures contribute to a higher pollen viability percentage. As the correlation matrix is a result of mathematical inference, all parameters cannot be assumed to be correctly correlated, as the physiological functions of the parameters also need to be considered. In this regard, the negative correlation obtained between certain parameters cannot be explained, as the physiological basis of their relationship is not possible.

### Inference of the evaluation of the F_2_ population

Based on the results of the phenotypic evaluation of the F_2_ population and their parents, we can characterize the NL-44 variety as a tolerant parent as it has performed better with regards to traits such as 1,000 grain weight, spikelet fertility, pollen viability, transpiration rate, photosynthetic rate, membrane stability index, and days to flower compared to the variety Uma under high temperature stress conditions. The poor performance of the Uma variety under stress conditions makes it a suitable candidate for characterization as a susceptible variety to heat stress. The mean of the population was close to NL-44 with regards to the traits of plant height, membrane stability index, photosynthetic rate, stomatal conductance, transpiration rate, pollen viability, spikelet fertility, and 1,000 grain weight. However, for traits such as tiller number, days to flower, time of anthesis, leaf temperature, and panicle length, the mean of the population was influenced by the susceptible parent, Uma. The yield-contributing characteristics, such as productive tiller number, have been depressed in the F_2_ population.

### Polymorphic markers related to high temperature tolerance

Simple sequence repeats (SSR) are molecular markers that are relatively more efficient, cheaper, and easier to use in marker assisted selection for crop improvement, as there is a probability of a higher polymorphism rate (Beena et al., 2012; Gao et al., 2016). Identifying SSR markers that are associated with QTLs contributing to heat tolerance has huge potential in breeding programs through gene pyramiding (Ye et al., 2015; Ali et al., 2022). In this regard, bulked segregant analysis is a technique that can rapidly identify markers that are tightly linked to genes for a given phenotype (Zou et al., 2016).

In the current study, out of the 100 SSR markers tested on the parental genotypes and the bulks, 18 were found to be polymorphic indicating a polymorphism of 18%. BSA was used to link the markers to heat tolerance based on spikelet fertility percentage recorded under stress conditions. Waghmare et al. (2018) had demonstrated the efficiency of BSA, through which they had identified 41 SSR markers that were found to be polymorphic between parents and associated with QTLs for heat tolerance. Varying levels of polymorphism between the tolerant and susceptible parents were obtained, ranging from 8.07% to 27.99%, in studies that had utilised SSR markers to link them with heat tolerance (Kanagaraj et al., 2010; Salunkhe et al., 2011; Vikram et al., 2011; Wei et al., 2013).

Although a good number of markers linked to different traits for tolerance to heat stress have been reported, each segregated population is unique due to genetic differences between the parents, and therefore, the markers validated in a particular cross of parental genotypes may not be applicable to other populations. In the present study, a significant proportion of previously reported validated markers for heat tolerance were used, but only a few of them could be confirmed with the results we have obtained. Out of the 18 polymorphic markers identified, only one (RM337) is expected to be unique to the current investigation, and it has not been reported in the literature surveyed. Among the 18 polymorphic markers, the markers RM222, RM237, RM556, and RM3475 have also been linked to drought tolerance traits (Yue et al., 2006; Freeg et al., 2016).

The rest of the identified polymorphic markers have been reported to be linked to various aspects of heat tolerance. RM554 was reported to be associated with QTLs that controlled the unfilled grain percentage (Buu et al., 2014). RM3586 was validated to be associated with QTLs responsible for phenotypic variation in heat tolerance at the flowering stage (Zhang et al., 2009; Buu et al., 2014). Xiao et al. (2011) associated RM471 with QTLs influencing seed set percentage. The marker RM242 was reported to influence the erect panicle trait (Kong et al., 2007) and was also associated with plant height, panicle length, and spikelet fertility under heat stress by Wei et al. (2013); Pradhan et al. (2016), and Mohapatra et al., 2021. Sunoharaa et al., (2016) reported that RM3475 and RM237 were associated with controlling panicle characters. Identified RM473 as a polymorphic marker that was strongly associated with heat tolerance. RM473 was also found to be associated with the QTL *qEHD10*, that regulates the thermosensitive-linked heading date in rice (Kovi et al., 2015). Pradhan et al. (2016) had associated RM3586 with days to 50% flowering and spikelet fertility percentage under high temperature stress. Bharathkumar et al. (2014), in their study, found association between RM6100 and a major quantitative trait locus affecting tolerance to heat stress at the flowering stage. The association of RM6100 with high temperature tolerance in rice was also reported by Stephen et al. (2022a). Revathi et al. (2013) reported that RM6100 linked to the fertility restoration gene, Rf4, had 85% efficiency in identifying restoration lines in rice. Waghmare et al. (2021) identified RM5749 as a polymorphic marker that could differentiate between the tolerant and susceptible bulks under heat stress in a cross of the parental genotypes, N22 and Uma. RM222 was noted to be 11.8 cM from the AFLP marker Rev1 that was linked to the recessive thermogenic male sterility (tgms) associated gene, i.e., *rtms1* (Jia et al., 2001). However, the direct association of RM222 with the quantitative character of heat tolerance has not been widely studied. Aliyu et al. (2011) validated the association of RM10793 with the QTL *SALTOL*, which indicates its association with multiple abiotic stress factors.

Through QTL analysis, the identified polymorphic markers can be linked to traits known to contribute to heat tolerance. The polymorphic markers are useful in identifying marker loci associated with various phenotypic traits. In the studied F_2_ population, the plants were segregated into tolerant or susceptible genotypes based on the spikelet fertility percentage alone. Therefore, the markers may or may not be linked to other phenotypic traits that were used for evaluation. However, an inference can be made from the correlation analysis between the traits, wherein the spikelet fertility percentage was significantly and positively associated with tolerance and yield contributing traits such as pollen viability, MSI, and 1,000 grain weight. The markers can be utilized for marker-assisted selection to incorporate tolerance traits. As some of the markers have been reported to be associated with drought, further studies are needed to identify markers that are specific to heat tolerance. These studies will be used to identify traits that may be useful for such characterization.

The identified polymorphic markers have been further investigated using bioinformatics tools to annotate genes associated with them. The genomic regions in which the markers are present have been found to encode several important genes that are involved in varied metabolic, structural, and stress-related functions. The annotation of such genes provides a clue to the mechanisms or pathways that might involve their plant functions. For instance, the gene Snf-7 (Sucrose non-fermenting 7) family protein, which is involved in regulating sucrose metabolism under stress conditions, has been found to be associated with the marker RM10793. Apart from this, genes for nitrate transporter and nitrogen-use efficiency, as well as the NAC transcription factor protein, which regulates the synthesis of starch and storage proteins, have been annotated, among several other critical genes. The marker RM473 was found to be associated with the gene GPI (Glycophosphatidylinositol)-anchored membrane protein, which regulates leaf rolling and maintains epidermal integrity, cell wall formation, and homeostasis, as well as the gene SLG-7-like protein, which regulates the slender grain shape formation. Expression analysis of the genes *LOP1* (LOC_Os08g01330) and *LOP2* **(**LOC_Os08g0112) associated with the marker RM337 revealed that *LOP1* was linked with high temperature tolerance in rice as per the phenotypic evaluation. The diverse functions of the genes indicate their involvement in the regulation of stress responses.

## Conclusion

The phenotyping of the F_2_ generation indicated that the tolerance traits in the population were majorly contributed by the tolerant parent, i.e., NL-44. The identified polymorphic markers were able to segregate the individual lines of the F_2_ population into tolerant and susceptible genotypes. The association of molecular markers linked to the heat tolerance traits in the segregating second-generation filial populations is validated to be beneficial in undertaking crop improvement studies to introgress the tolerance traits into high-yielding regional varieties. Most of the genes annotated are yet to be exploited for their beneficial effects. Further studies are required to link them specifically to the tolerance traits. The genomic areas associated with heat tolerance traits should be further investigated or fine-mapped to discover potential genes or putative QTLs controlling the pathways underpinning heat tolerance traits for marker-assisted selection programs.

## Data availability statement

The original contributions presented in the study are included in the article/**Supplementary Material**. Further inquiries can be directed to the corresponding author.

## Author contributions

RB conceptualized the study. KS, KA and RB performed the work. Data analysis was done by KS, KA RPS, BS and UCJ. All authors reviewed and approved the manuscript.
