# Supplementary material for: Identification of simple sequence repeat markers linked to heat tolerance in rice using bulked segregant analysis in F2 population of NERICA-L 44 × Uma
Source: Front Plant Sci. 2023 Mar 27;14:1113838. doi: 10.3389/fpls.2023.1113838 (PMC10084929; doi:10.3389/fpls.2023.1113838)
Supplement: Supplementary file 1 [file DataSheet_1.doc]

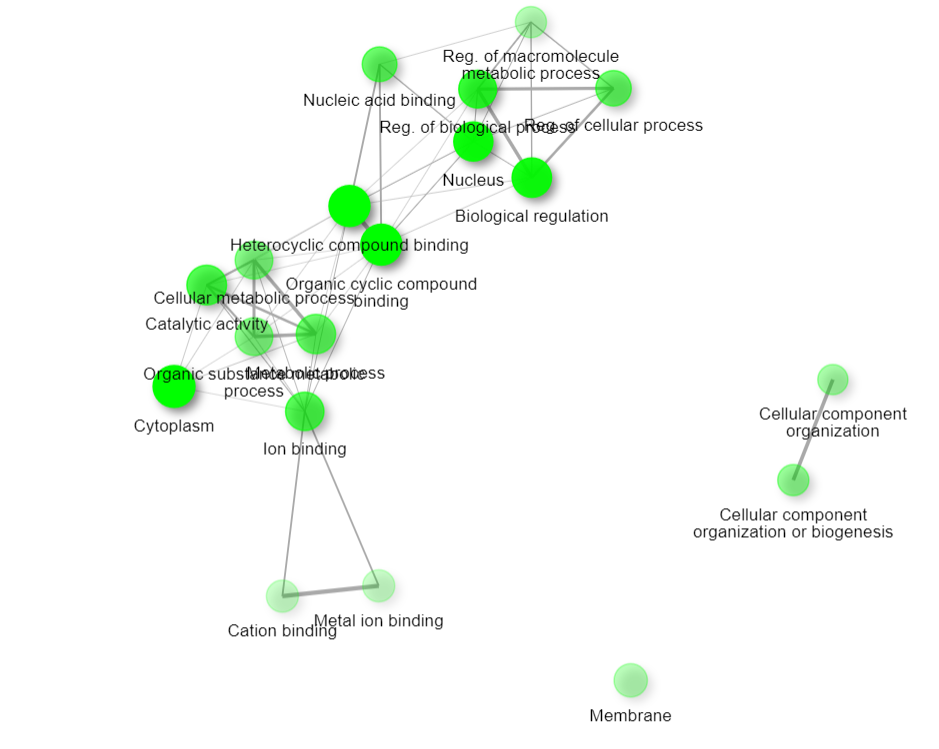


**Supplementary Figure 1: Network map of functions of the annotated genes associated with the marker RM10793**


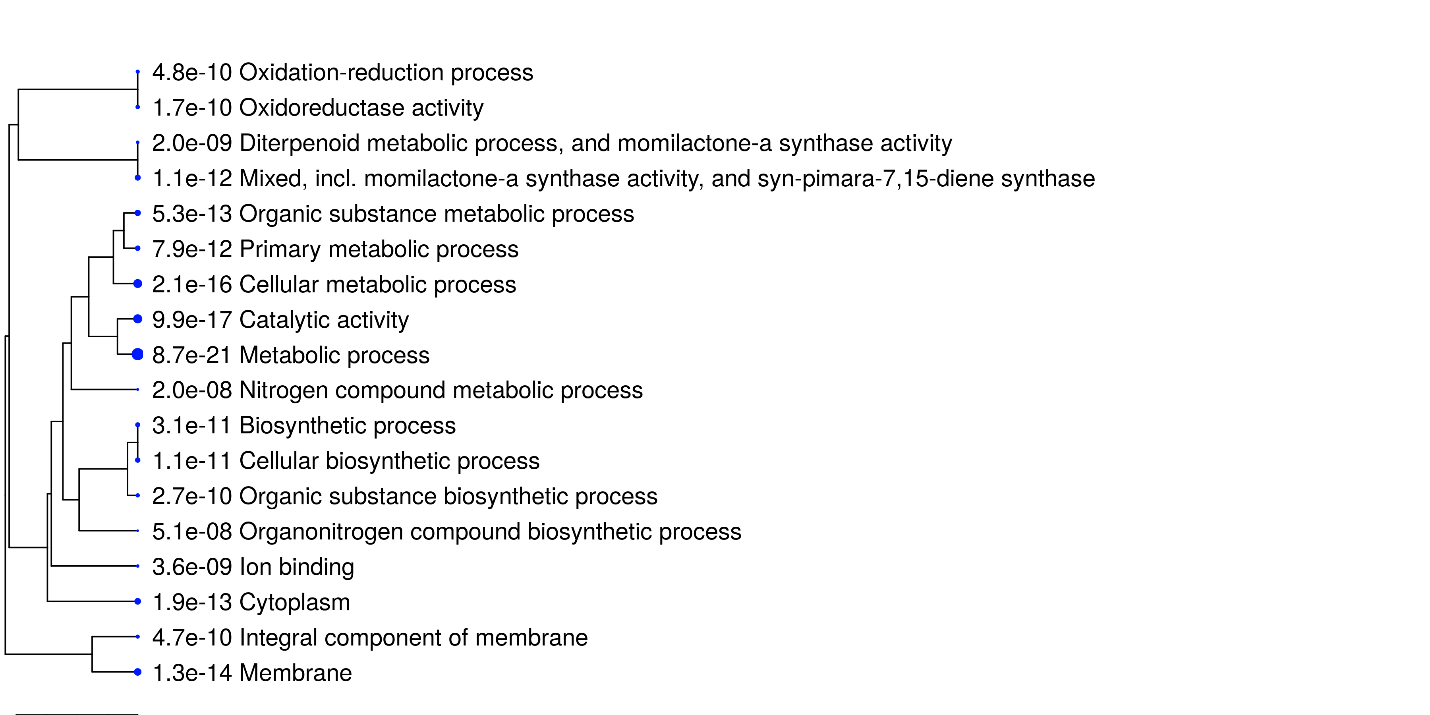


**Supplementary Figure 2: Tree-diagram of the diverse cellular functions of the genes associated with RM5749**


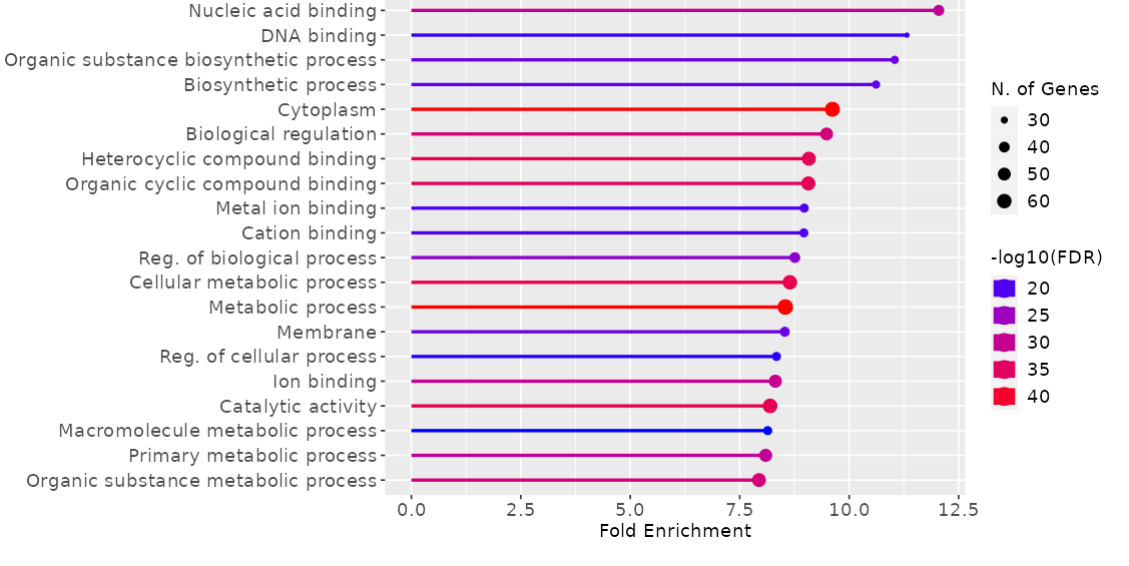
**Supplementary Figure 3: Bar-plot (RM473) representing the number of genes involved in the major cellular functions**
