# Supplementary material for: Identification of simple sequence repeat markers linked to heat tolerance in rice using bulked segregant analysis in F2 population of NERICA-L 44 × Uma
Source: Front Plant Sci. 2023 Mar 27;14:1113838. doi: 10.3389/fpls.2023.1113838 (PMC10084929; doi:10.3389/fpls.2023.1113838)
Supplement: Supplementary file 2 [file Table_1.doc]

**Supplementary table 1: Annotation - Gene annotation and trait ontology of 9 RM markers identified in BSA for heat tolerance**

| **Sl no** | **Marker** | **Loci** | **Description** | **Chromosome No.** | **Gene name** | **Gene symbol** | **Trait Ontology** |
| --- | --- | --- | --- | --- | --- | --- | --- |
| 1 | RM490 | LOC_Os01g04350 | Similar to Cytosolic class I small heat shock protein 3C (Fragment). (Os01t0135900-01);LMW heat shock protein. (Os01t0135900-02) | 01 | NA | NA | TO:0000259 - heat tolerance |
|  |  | LOC_Os01g04360 | 16.9 kDa small heat shock protein (Os01t0136000-01) | 01 | OsHSP16.9C | NA | TO:0000259 - heat tolerance |
|  |  | LOC_Os01g04380 | 16.9 kDa class I heat shock protein 1. (Os01t0136200-01) | 01 | NA | NA | TO:0000259 - heat tolerance |
|  |  | LOC_Os01g07120 | Transcription factor, Dehydration and salt stress tolerance (Os01t0165000-01);DRE binding protein 2. (Os01t0165000-02) | 01 | DREB2A, OsDREB2A | DEHYDRATION-RESPONSIVE ELEMENT-BINDING PROTEIN 2A, Dehydration-responsive element-binding protein 2A | TO:0000259 - heat tolerance,TO:0000276 - drought tolerance,TO:0006001 - salt tolerance,TO:0000615 - abscisic acid sensitivity,TO:0000303 - cold tolerance,TO:0000058 - herbicide sensitivity |
|  |  | LOC_Os01g07810 | Alba, DNA/RNA-binding protein family protein. (Os01t0173100-01) | 01 | NA | NA | TO:0000172 - jasmonic acid sensitivity,TO:0000615 - abscisic acid sensitivity,TO:0000303 - cold tolerance,TO:0000259 - heat tolerance,TO:0006001 - salt tolerance,TO:0000276 - drought tolerance |
|  |  | LOC_Os01g08860 | Class II small heat shock protein, Positive regulation in both Xoo resistance and heat/salt tolerance (Os01t0184100-01) | 01 | OsHsp18.0, Oshsp18.0-CII | Oryza sativa small heat shock protein 18.0 | TO:0000175 - bacterial blight disease resistance,TO:0000435 - seed longevity,TO:0002661 - seed maturation,TO:0000430 - germination rate,TO:0000259 - heat tolerance,TO:0006001 - salt tolerance,TO:0000250 - vigor related trait |
|  |  | LOC_Os01g09020 | WD40 repeat domain containing protein. (Os01t0185400-01);Substrate receptor of Cullin4-based E3 ubiquitin ligase complex (C4E3), Regulation of abiotic stress response and productivity (Os01t0185400-02) | 01 | OsCBE1 | Cullin4-Based E3 ubiquitin ligase1 | TO:0000152 - panicle number,TO:0000276 - drought tolerance,TO:0000615 - abscisic acid sensitivity,TO:0002759 - grain number,TO:0006001 - salt tolerance,TO:0000316 - photosynthetic ability,TO:0000303 - cold tolerance,TO:0001016 - relative chlorophyll content |
|  |  | LOC_Os01g09100 | WRKY transcription factor 10, Negative regulation of thermotolerance | 01 | WRKY10 | WRKY transcription factor 10 | TO:0000175 - bacterial blight disease resistance,TO:0000303 - cold tolerance,TO:0000276 - drought tolerance,TO:0000259 - heat tolerance,TO:0006001 - salt tolerance,TO:0000249 - leaf senescence,TO:0000615 - abscisic acid sensitivity |
|  |  | LOC_Os01g11860 | ThiJ/PfpI domain containing protein. (Os01t0217500-01) | 01 | NA | NA | TO:0000080 - micronutrient sensitivity,TO:0000303 - cold tolerance,TO:0000259 - heat tolerance,TO:0000439 - fungal disease resistance,TO:0000021 - copper sensitivity,TO:0002657 - oxidative stress |
| 2 | RM10793 | LOC_Os01g08700 | Orthologue of the Arabidopsis GIGANTEA, Regulation of circadian rhythm and flowering time, Photoperiodic control of flowering, Osmotic stress response (Os01t0182600-01);GIGANTEA protein. (Os01t0182600-03) | 01 | GI, OsGI | GIGANTEA | TO:0000075 - light sensitivity,TO:0000432 - temperature response trait,TO:0002616 - flowering time,TO:0000137 - days to heading,TO:0000095 - osmotic response sensitivity,TO:0000328 - sucrose content,TO:0006002 - proline content,TO:0001018 - transpiration rate,TO:0000523 - stomatal resistance,TO:0000278 - root to shoot ratio,TO:0000227 - root length,TO:0000276 - drought tolerance |
|  |  | LOC_Os01g04350 | Similar to Cytosolic class I small heat shock protein 3C (Fragment). (Os01t0135900-01);LMW heat shock protein. (Os01t0135900-02) | 01 | NA | NA | TO:0000259 - heat tolerance |
|  |  | LOC_Os01g04360 | 16.9 kDa small heat shock protein (Os01t0136000-01) | 01 | OsHSP16.9C | NA | TO:0000259 - heat tolerance |
|  |  | LOC_Os01g04380 | 16.9 kDa class I heat shock protein 1. (Os01t0136200-01) | 01 | NA | NA | TO:0000259 - heat tolerance |
|  |  | LOC_Os01g04730 | Similar to 60S ribosomal protein L26B. (Os01t0140500-01) | 01 | NA | NA | TO:0000175 - bacterial blight disease resistance,TO:0000303 - cold tolerance,TO:0002657 - oxidative stress,TO:0000259 - heat tolerance,TO:0000172 - jasmonic acid sensitivity,TO:0000276 - drought tolerance |
|  |  | LOC_Os01g07120 | Transcription factor, Dehydration and salt stress tolerance (Os01t0165000-01);DRE binding protein 2. (Os01t0165000-02) | 01 | DREB2A, OsDREB2A | DEHYDRATION-RESPONSIVE ELEMENT-BINDING PROTEIN 2A, Dehydration-responsive element-binding protein 2A | TO:0000259 - heat tolerance,TO:0000276 - drought tolerance,TO:0006001 - salt tolerance,TO:0000615 - abscisic acid sensitivity,TO:0000303 - cold tolerance,TO:0000058 - herbicide sensitivity |
|  |  | LOC_Os01g07810 | Alba, DNA/RNA-binding protein family protein. | 01 | NA | NA | TO:0000172 - jasmonic acid sensitivity,TO:0000615 - abscisic acid sensitivity,TO:0000303 - cold tolerance,TO:0000259 - heat tolerance,TO:0006001 - salt tolerance,TO:0000276 - drought tolerance |
|  |  | LOC_Os01g08860 | Class II small heat shock protein, Positive regulation in both Xoo resistance and heat/salt tolerance | 01 | OsHsp18.0, Oshsp18.0-CII | Oryza sativa small heat shock protein 18.0 | TO:0000175 - bacterial blight disease resistance,TO:0000435 - seed longevity,TO:0002661 - seed maturation,TO:0000430 - germination rate,TO:0000259 - heat tolerance,TO:0006001 - salt tolerance,TO:0000250 - vigor related trait |
|  |  | LOC_Os01g09100 | WRKY transcription factor 10, Negative regulation of thermotolerance | 01 | WRKY10 | WRKY transcription factor 10 | TO:0000175 - bacterial blight disease resistance,TO:0000303 - cold tolerance,TO:0000276 - drought tolerance,TO:0000259 - heat tolerance,TO:0006001 - salt tolerance,TO:0000249 - leaf senescence,TO:0000615 - abscisic acid sensitivity |
|  |  | LOC_Os01g11860 | ThiJ/PfpI domain containing protein. (Os01t0217500-01) | 01 | NA | NA | TO:0000080 - micronutrient sensitivity,TO:0000303 - cold tolerance,TO:0000259 - heat tolerance,TO:0000439 - fungal disease resistance,TO:0000021 - copper sensitivity,TO:0002657 - oxidative stress |
|  |  | LOC_Os01g13540 | Nodule inception (NIN) like protein 3, Regulation of nitrogen use efficiency and grain yield under nitrate-sufficient conditions (Os01t0236700-01) | 01 | OsNLP3 | NIN LIKE PROTEIN 3, NIN like protein 3 | TO:0000276 - drought tolerance,TO:0000615 - abscisic acid sensitivity,TO:0000011 - nitrogen sensitivity,TO:0000167 - cytokinin sensitivity,TO:0000303 - cold tolerance,TO:0000259 - heat tolerance |
|  |  | LOC_Os01g14440 | Similar to WRKY transcription factor 1. (Os01t0246700-01) | 01 | NA | NA | TO:0000303 - cold tolerance,TO:0000259 - heat tolerance,TO:0000615 - abscisic acid sensitivity,TO:0006001 - salt tolerance,TO:0000175 - bacterial blight disease resistance,TO:0000276 - drought tolerance |
|  |  | LOC_Os01g15640 | NAC transcription factor, Heat stress tolerance (Os01t0261200-01);Similar to NAC domain-containing protein 74. (Os01t0261200-02) | 01 | OsNTL3 | NA | TO:0000259 - heat tolerance,TO:0000303 - cold tolerance,TO:0000276 - drought tolerance,TO:0006001 - salt tolerance,TO:0000655 - leaf development trait |
|  |  | LOC_Os01g16220 | Sad1/UNC-84 (SUN) domain protein, Promotion of telomere clustering and homologous pairing in meiosis (Os01t0267600-01) | 01 | OsSUN2 | Sad1/UNC-84 (SUN) domain protein 2 | TO:0000615 - abscisic acid sensitivity,TO:0000259 - heat tolerance,TO:0006001 - salt tolerance |
|  |  | LOC_Os01g16970 | Glucosidase II β subunit, Potential switch between programmed cell death and autophagy (Os01t0276800-01);Similar to predicted protein. (Os01t0276800-02);Hypothetical conserved gene. (Os01t0276800-03);Similar to predicted protein. (Os01t0276800-04);Similar to predicted protein. (Os01t0276800-05) | 01 | Gas2, OsGAS2 | glucosidase II β subunit, glucosidase II b subunit | TO:0000303 - cold tolerance,TO:0000259 - heat tolerance,TO:0000095 - osmotic response sensitivity,TO:0006001 - salt tolerance |
|  |  | LOC_Os01g17260 | TGA transcription factor, bZIP transcription factor, Defense response, Multiple abiotic stress tolerance (Os01t0279900-01) | 01 | OsTGA5, OsbZIP3, OsHBP1b | Histone gene binding protein-1b | TO:0000605 - hydrogen peroxide content,TO:0000259 - heat tolerance,TO:0000175 - bacterial blight disease resistance,TO:0006001 - salt tolerance,TO:0000495 - chlorophyll content,TO:0000615 - abscisic acid sensitivity,TO:0000316 - photosynthetic ability,TO:0000276 - drought tolerance,TO:0000172 - jasmonic acid sensitivity,TO:0000112 - disease resistance |
|  |  | LOC_Os01g18080 | Conserved hypothetical protein. (Os01t0283300-01);Conserved hypothetical protein. (Os01t0283300-02) | 01 | NA | NA | TO:0000259 - heat tolerance |
|  |  | LOC_Os01g18170 | Similar to Nectarin 1 precursor (EC 1.15.1.1) (Superoxide dismutase [Mn]). (Os01t0284500-01) | 01 | NA | NA | TO:0000315 - bacterial disease resistance,TO:0000303 - cold tolerance,TO:0000276 - drought tolerance,TO:0000259 - heat tolerance,TO:0000439 - fungal disease resistance |
|  |  | LOC_Os01g08700 | Orthologue of the Arabidopsis GIGANTEA, Regulation of circadian rhythm and flowering time, Photoperiodic control of flowering, Osmotic stress response (Os01t0182600-01);GIGANTEA protein. (Os01t0182600-03) | [01](https://rapdb.dna.affrc.go.jp/jbrowse/?data=data%2Firgsp1&loc=chr01%3A4327080.75..4340767.25&highlight=chr01%3A4329362..4338486) | GI, OsGI | GIGANTEA | TO:0000075 - light sensitivity,TO:0000432 - temperature response trait,TO:0002616 - flowering time,TO:0000137 - days to heading,TO:0000095 - osmotic response sensitivity,TO:0000328 - sucrose content,TO:0006002 - proline content,TO:0001018 - transpiration rate,TO:0000523 - stomatal resistance,TO:0000278 - root to shoot ratio,TO:0000227 - root length,TO:0000276 - drought tolerance |
| 3 | RM470 | LOC_Os04g01740 | Similar to Heat shock protein 82. (Os04t0107900-01);Heat shock protein 81-1 (HSP81-1) (Heat shock protein 83). (Os04t0107900-02);Similar to Heat shock protein 80. (Os04t0107900-03);Similar to Heat shock protein 82. (Os04t0107900-04);Non-protein coding transcript. (Os04t0107900-05) | 04 | NA | NA | TO:0000259 - heat tolerance,TO:0000303 - cold tolerance |
|  |  | LOC_Os04g05010 | Cystathionine beta-synthase, core domain containing protein. (Os04t0136700-01) | 04 | NA | NA | TO:0000259 - heat tolerance |
|  |  | LOC_Os04g21350 | Homologue of Arabidopsis flowering-promoting factor 1, AtFPF1, Modulation of root and flower development, Control of auxin accumulation, Modulation of ROS homeostasis (Os04t0282400-01) | 04 | OsFPFL4 | FPF1-like protein 4, flowering-promoting factor 1-like protein 4 | TO:0006001 - salt tolerance,TO:0000276 - drought tolerance,TO:0000095 - osmotic response sensitivity,TO:0000259 - heat tolerance,TO:0000303 - cold tolerance,TO:0000163 - auxin sensitivity,TO:0000656 - root development trait,TO:0002616 - flowering time,TO:0001006 - adventitious root number,TO:0001013 - lateral root number,TO:0000227 - root length,TO:0000605 - hydrogen peroxide content,TO:0002672 - auxin content |
|  |  | LOC_Os04g26910 | Similar to IN2-2 protein. (Os04t0338000-01) | 04 | NA | NA | TO:0000259 - heat tolerance |
|  |  | LOC_Os04g28420 | FK506-binding protein, Peptidyl-prolyl cis/trans isomerase, Chilling tolerance (Os04t0352400-01) | 04 | OsFKBP65, OsFKBP62b | FK506-binding protein 65 | TO:0000303 - cold tolerance,TO:0000259 - heat tolerance,TO:0002657 - oxidative stress |
|  |  | LOC_Os04g32950 | Similar to Calnexin (Fragment). (Os04t0402100-01);Similar to calnexin. (Os04t0402100-02) | 04 | NA | NA | TO:0000615 - abscisic acid sensitivity,TO:0000259 - heat tolerance,TO:0000303 - cold tolerance,TO:0000276 - drought tolerance,TO:0006001 - salt tolerance |
|  |  | LOC_Os04g33210 | Similar to ATP-dependent Clp protease ATP-binding subunit. (Os04t0405000-01) | 04 | NA | NA | TO:0000303 - cold tolerance,TO:0000259 - heat tolerance,TO:0002657 - oxidative stress |
|  |  | LOC_Os04g36750 | Similar to 22.7 kDa class IV heat shock protein precursor. (Os04t0445100-01) | 04 | NA | NA | TO:0000259 - heat tolerance |
|  |  | LOC_Os04g37619 | Zeaxanthin epoxidase, Abscisic acid (ABA) biosynthesis, "Resistance to osmotic and drought stresses, seed development and dormancy", Disease resistance (Os04t0448900-01) | 04 | OsABA1, OsZEP | abscisic acid-deficient 1, zeaxanthin epoxidase | TO:0000478 - abscisic acid concentration,TO:0000326 - leaf color,TO:0000615 - abscisic acid sensitivity,TO:0000148 - viral disease resistance,TO:0000020 - black streak dwarf virus resistance,TO:0000203 - bacterial leaf streak disease resistance,TO:0000131 - leaf water potential,TO:0000175 - bacterial blight disease resistance,TO:0000619 - vivipary,TO:0006001 - salt tolerance,TO:0000522 - stomatal conductance,TO:0002667 - abscisic acid content,TO:0000432 - temperature response trait,TO:0000227 - root length,TO:0000207 - plant height,TO:0000135 - leaf length,TO:0000276 - drought tolerance,TO:0000259 - heat tolerance |
|  |  | LOC_Os04g39020 | Betaine aldehyde dehydrogenase, Rice fragrance, Salt stress (Os04t0464200-01) | 04 | BAD1, Badh1, badh1, BADH1, OsBADH1, BADH | BETAINE ALDEHYDE DEHYDROGENASE 1, betaine aldehyde dehydrogenase 1 | TO:0000303 - cold tolerance,TO:0000259 - heat tolerance,TO:0000286 - submergence sensitivity,TO:0006001 - salt tolerance,TO:0000276 - drought tolerance,TO:0000075 - light sensitivity |
|  |  | LOC_Os04g39100 | Haem peroxidase, plant/fungal/bacterial family protein. (Os04t0465100-01) | 04 | NA | NA | TO:0000259 - heat tolerance |
|  |  | LOC_Os04g39700 | Ribosomal protein large subunit member, Salt stress tolerance (Os04t0473400-01) | 04 | RPL6 | Ribosomal Protein L6, Ribosomal Protein Large subunit 6 | TO:0000172 - jasmonic acid sensitivity,TO:0000276 - drought tolerance,TO:0002657 - oxidative stress,TO:0000175 - bacterial blight disease resistance,TO:0000303 - cold tolerance,TO:0000259 - heat tolerance |
|  |  | LOC_Os04g41850 | NIN-like protein 2, Nitrate responsive transcript factor, Regulation of ABA content during seed germination under salt stress (Os04t0495800-01);Similar to OSIGBa0159F11.11 protein. (Os04t0495800-02) | 04 | OsNLP2 | NIN-like protein 2 | TO:0000259 - heat tolerance,TO:0000276 - drought tolerance,TO:0000303 - cold tolerance,TO:0000167 - cytokinin sensitivity,TO:0000615 - abscisic acid sensitivity |
|  |  | LOC_Os04g45480 | Tetratricopeptide TPR-1 domain containing protein. (Os04t0538000-01);Hypothetical gene. (Os04t0538000-02) | 04 | NA | NA | TO:0000074 - blast disease,TO:0000259 - heat tolerance |
|  |  | LOC_Os04g46060 | Transcriptional repressor for secondary cell wall formation, Repression of rice lignification, Negative regulation of plant height and grain size, Inhibition of gibberellin signaling (Os04t0545000-01) | 04 | OsWRKY36, SGSD3 | WRKY GENE 36, small grain and semi-dwarf 3 | TO:0000175 - bacterial blight disease resistance,TO:0000259 - heat tolerance |
|  |  | LOC_Os04g46830 | Similar to RCc3 protein. | 04 | NA | NA | TO:0000259 - heat tolerance,TO:0000615 - abscisic acid sensitivity,TO:0006001 - salt tolerance,TO:0000303 - cold tolerance,TO:0000074 - blast disease,TO:0000172 - jasmonic acid sensitivity |
| 4 | RM5749 | LOC_Os04g01740 | Similar to Heat shock protein 82. (Os04t0107900-01);Heat shock protein 81-1 (HSP81-1) (Heat shock protein 83). (Os04t0107900-02);Similar to Heat shock protein 80. (Os04t0107900-03);Similar to Heat shock protein 82. (Os04t0107900-04);Non-protein coding transcript. (Os04t0107900-05) | 04 | NA | NA | TO:0000259 - heat tolerance,TO:0000303 - cold tolerance |
|  |  | LOC_Os04g05010 | Cystathionine beta-synthase, core domain containing protein. (Os04t0136700-01) | 04 | NA | NA | TO:0000259 - heat tolerance |
|  |  | LOC_Os04g21350 | Homologue of Arabidopsis flowering-promoting factor 1, AtFPF1, Modulation of root and flower development, Control of auxin accumulation, Modulation of ROS homeostasis (Os04t0282400-01) | 04 | OsFPFL4 | FPF1-like protein 4, flowering-promoting factor 1-like protein 4 | TO:0006001 - salt tolerance,TO:0000276 - drought tolerance,TO:0000095 - osmotic response sensitivity,TO:0000259 - heat tolerance,TO:0000303 - cold tolerance,TO:0000163 - auxin sensitivity,TO:0000656 - root development trait,TO:0002616 - flowering time,TO:0001006 - adventitious root number,TO:0001013 - lateral root number,TO:0000227 - root length,TO:0000605 - hydrogen peroxide content,TO:0002672 - auxin content |
|  |  | LOC_Os04g26910 | Similar to IN2-2 protein. (Os04t0338000-01) | 04 | NA | NA | TO:0000259 - heat tolerance |
|  |  | LOC_Os04g28420 | FK506-binding protein, Peptidyl-prolyl cis/trans isomerase, Chilling tolerance (Os04t0352400-01) | 04 | OsFKBP65, OsFKBP62b | FK506-binding protein 65 | TO:0000303 - cold tolerance,TO:0000259 - heat tolerance,TO:0002657 - oxidative stress |
| 5 | RM473 | LOC_Os07g04230 | Mitochodrial transcription termination factor-related family protein. (Os07t0134700-00) | 07 | NA | NA | TO:0000259 - heat tolerance |
|  |  | LOC_Os07g05360 | Similar to Photosystem II 10 kDa polypeptide, chloroplast precursor. (Os07t0147500-01) | 07 | NA | NA | TO:0000615 - abscisic acid sensitivity,TO:0006001 - salt tolerance,TO:0000276 - drought tolerance,TO:0000259 - heat tolerance,TO:0000303 - cold tolerance |
|  |  | LOC_Os07g07350 | A20/AN1-type zinc finger protein, Response to various abiotic stresses, Temperature stress tolerance (Os07t0168800-01) | 07 | ZFP177 | ZINC FINGER PROTEIN 177 | TO:0000303 - cold tolerance,TO:0006001 - salt tolerance,TO:0000164 - stress trait,TO:0000276 - drought tolerance,TO:0000259 - heat tolerance |
|  |  | LOC_Os07g11910 | Prolamin precursor (13 kDa prolamin). (Os07t0219300-01) | 07 | NA | NA | TO:0000259 - heat tolerance |
|  |  | LOC_Os07g33898 | Similar to 60S ribosomal protein L44. (Os07t0523300-01);Similar to 60S ribosomal protein L44. (Os07t0523300-02);Non-protein coding transcript. (Os07t0523300-03) | 07 | NA | NA | TO:0000259 - heat tolerance,TO:0002657 - oxidative stress,TO:0000172 - jasmonic acid sensitivity,TO:0000303 - cold tolerance,TO:0000276 - drought tolerance |
| 6 | RM337 | LOC_Os08g01090 | ABI3/VP1 transcription factor family protein, Regulation of iron-deficiency response and tolerance (Os08t0101000-01) | 08 | IDEF1, OsLFL2, OsIDEF1 | IDE-BINDING FACTOR 1, B3 domain-containing protein IDEF1, Protein IRON DEFICIENCY-RESPONSIVE ELEMENT FACTOR 1, Transcription factor IDEF1, LEAFY COTYLEDON 2 and FUSCA 3-LIKE 2, IDE-binding factor 1, Iron Deficiency-responsive Element-binding Factor 1, iron deficiency-responsive cis-acting element binding factor 1 | TO:0000495 - chlorophyll content,TO:0000615 - abscisic acid sensitivity,TO:0000207 - plant height,TO:0000224 - iron sensitivity,TO:0000172 - jasmonic acid sensitivity |
|  |  | LOC_Os08g01120 | Molybdate transporter, Uptake and translocation of molybdate (Os08t0101500-01) | 08 | OsMOT1;1 | molybdate transporter 1;1 | TO:0000276 - drought tolerance,TO:0000653 - seed development trait,TO:0000025 - molybdenum sensitivity,TO:0000034 - chromium sensitivity,TO:0000303 - cold tolerance,TO:0006001 - salt tolerance |
|  |  | LOC_Os08g01330 | NAC transcription factor, Regulation of cellulose synthesis, Regulation of secondary wall biosynthesis (Os08t0103900-01) | 08 | NAC31, OsSWN3 | secondary wall NAC transcription factor 3, secondary wall-associated NAC domain protein 3 | TO:0000621 - inflorescence development trait |
| 7 | RM242 | LOC_Os09g04790 | Fibrillin, Plastoglobule (PG) formation and lipid metabolism in chloroplasts, Heat stress response (Os09t0133600-01);PAP fibrillin family protein. (Os09t0133600-02) | 09 | OsFBN1 | fibrillin 1 | TO:0000448 - filled grain percentage,TO:0000259 - heat tolerance,TO:0000152 - panicle number,TO:0000346 - tiller number,TO:0002668 - jasmonic acid content |
|  |  | LOC_Os09g08430 | Similar to 60S ribosomal protein L17-1. (Os09t0258600-01) | 09 | NA | NA | TO:0002657 - oxidative stress,TO:0000172 - jasmonic acid sensitivity,TO:0000175 - bacterial blight disease resistance,TO:0000259 - heat tolerance,TO:0000276 - drought tolerance,TO:0000303 - cold tolerance |
|  |  | LOC_Os09g25760 | Similar to senescence-associated protein DH. (Os09t0425900-01);Similar to Senescence-associated protein 5. (Os09t0425900-02) | 09 | NA | NA | TO:0002657 - oxidative stress,TO:0000259 - heat tolerance,TO:0000095 - osmotic response sensitivity,TO:0000249 - leaf senescence,TO:0006001 - salt tolerance,TO:0000303 - cold tolerance |
|  |  | LOC_Os09g27750 | ACC oxidase, Ethylene biosynthesis (Os09t0451000-01) | 09 | ACO1, OsACO1, ACO | AMINOCYCLOPROPANE-1-CARBOXYLIC ACID OXIDASE 1, ACC oxidase 1, ACC oxidase | TO:0000259 - heat tolerance,TO:0000615 - abscisic acid sensitivity,TO:0000172 - jasmonic acid sensitivity,TO:0000276 - drought tolerance,TO:0000163 - auxin sensitivity |
|  |  | LOC_Os09g27830 | Similar to protein disulfide isomerase. (Os09t0451500-01);Protein disulfide isomerase (PDI) family oxidoreductase, Ortholog of human P5, Protein Body (PB) development in the endosperm, Storage protein biogenesis (Os09t0451500-02) | 09 | PDIL2;3 | Protein disulfide isomerase-like 2;3 | TO:0000259 - heat tolerance |
|  |  | LOC_Os09g28420 | Alpha-amylase 3C (Os09t0457800-01) | 09 | Amy3C | α-amylase 3C | TO:0000259 - heat tolerance,TO:0000166 - gibberellic acid sensitivity |
|  |  | LOC_Os09g29630 | mRNA 5-methylcytosine (m5C) methyltransferase, Adaptation to high temperature (Os09t0471900-01);Hypothetical conserved gene. (Os09t0471900-02) | 09 | OsNSUN2 | NOP2/Sun 2 | TO:0000259 - heat tolerance,TO:0000075 - light sensitivity,TO:0000063 - mimic response,TO:0000316 - photosynthetic ability,TO:0000303 - cold tolerance,TO:0000605 - hydrogen peroxide content,TO:0000227 - root length |
| 8 | RM222 | LOC_Os10g01060 | Serine/threonine protein kinase-related domain containing protein. (Os10t0100500-01);Similar to predicted protein. (Os10t0100500-02) | 10 | NA | NA | TO:0000621 - inflorescence development trait,TO:0000653 - seed development trait |
|  |  | LOC_Os10g02360 | Serine/threonine protein kinase-related domain containing protein. (Os10t0112700-01) | 10 | NA | NA | TO:0000175 - bacterial blight disease resistance,TO:0000074 - blast disease,TO:0000621 - inflorescence development trait,TO:0000653 - seed development trait |
|  |  | LOC_Os10g02584 | GAGA-binding transcription factor 3, Regulation of plant growth and grain size (Os10t0115200-01) | 10 | OsGBP3 | GAGA-binding transcription factor 3 | TO:0000207 - plant height,TO:0000734 - grain length |
|  |  | LOC_Os10g02650 | Pentatricopeptide repeat domain containing protein. (Os10t0116000-00) | 10 | NA | NA | TO:0000621 - inflorescence development trait |
|  |  | LOC_Os10g02770 | Similar to predicted protein. (Os10t0117000-01) | 10 | NA | NA | NA |
|  |  | LOC_Os10g02814 | Extra-large GTP-binding protein (Os10t0117800-01);Similar to cDNA clone:002-148-H04, full insert sequence. (Os10t0117800-02) | 10 | OsXLG3 | extra-large G protein 3 | TO:0000303 - cold tolerance,TO:0006001 - salt tolerance,TO:0000137 - days to heading,TO:0000276 - drought tolerance |
|  |  | LOC_Os10g02980 | Actin-binding FH2 domain containing protein. (Os10t0119300-01) | 10 | NA | NA | TO:0000146 - seed length,TO:0000391 - seed size,TO:0000207 - plant height,TO:0000043 - root anatomy and morphology trait,TO:0000547 - primary branch number |
|  |  | LOC_Os10g03660 | F-box domain, cyclin-like domain containing protein. (Os10t0125300-01);F-box protein, Tapetum cell development, Pollen formation, Control of anther development (Os10t0125300-02) | 10 | OsADF | anther development F-box | TO:0000187 - anther color,TO:0000421 - pollen fertility,TO:0000214 - anther shape,TO:0000531 - anther length |
| 9 | RM6100 | LOC_Os10g17260 | Flavonoid 3'-hydroxylase, Cytochrome P450 75B3, Flavone C-glycosides biosynthesis (Os10t0320100-01) | 10 | CYP75B3, F3'H | flavonoid 3'-hydroxylase | TO:0000071 - anthocyanin content,TO:0000653 - seed development trait,TO:0000259 - heat tolerance |
|  |  | LOC_Os10g25550 | Protein kinase, core domain containing protein. (Os10t0395000-01) | 10 | NA | NA | TO:0000259 - heat tolerance,TO:0000175 - bacterial blight disease resistance,TO:0000074 - blast disease,TO:0000621 - inflorescence development trait,TO:0000095 - osmotic response sensitivity,TO:0000653 - seed development trait,TO:0000172 - jasmonic acid sensitivity,TO:0000276 - drought tolerance |
|  |  | LOC_Os10g29180 | Similar to BTB/POZ domain containing protein, expressed. (Os10t0427300-01);Similar to BTB/POZ domain containing protein, expressed. (Os10t0427300-02) | Chr 10 | NA | NA | TO:0000259 - heat tolerance |
|  |  | LOC_Os10g29310 | Conserved hypothetical protein. (Os10t0428500-01) | 10 | NA | NA | TO:0000259 - heat tolerance |
|  |  | LOC_Os10g29495,LOC_Os10g29502 | Aminotransferase, class-II, pyridoxal-phosphate binding site domain containing protein. (Os10t0430600-01) | 10 | NA | NA | TO:0000259 - heat tolerance |
|  |  | LOC_Os10g33240 | Protein of unknown function DUF810 domain containing protein. (Os10t0471000-01) | 10 | NA | NA | TO:0000259 - heat tolerance,TO:0000615 - abscisic acid sensitivity,TO:0000303 - cold tolerance,TO:0000276 - drought tolerance,TO:0006001 - salt tolerance |
